# A comparative study of the superior longitudinal fasciculus subdivisions between neonates and young adults

**DOI:** 10.1007/s00429-022-02565-z

**Published:** 2022-09-17

**Authors:** Wenjia Liang, Qiaowen Yu, Wenjun Wang, Thijs Dhollander, Emmanuel Suluba, Zhuoran Li, Feifei Xu, Yang Hu, Yuchun Tang, Shuwei Liu

**Affiliations:** 1 Department of Anatomy and Neurobiology, Research Center for Sectional and Imaging Anatomy, Shandong Key Laboratory of Mental Disorders, Shandong Key Laboratory of Digital Human and Clinical Anatomy, School of Basic Medical Sciences, Cheeloo College of Medicine, Shandong University, Jinan, Shandong 250012 China; 2 Institute of Brain and Brain-Inspired Science, Shandong University, Jinan, Shandong 250012 China; 3 Department of Medical Imaging, Shandong Provincial Hospital Affiliated to Shandong First Medical University, Jinan, Shandong 250021 China; 4 Developmental Imaging, Murdoch Children’s Research Institute, Melbourne, Victoria Australia; 5 Department of Ultrasound, Shandong Provincial Hospital Affiliated to Shandong First Medical University, Jinan, Shandong 250021 China

**Keywords:** Neonatal brain, NODDI, Superior longitudinal fasciculus, Fibre tractography

## Abstract

The superior longitudinal fasciculus (SLF) is a complex associative tract comprising three distinct subdivisions in the frontoparietal cortex, each of which has its own anatomical connectivity and functional roles. However, many studies on white matter development, hampered by limitations of data quality and tractography methods, treated the SLF as a single entity. The exact anatomical trajectory and developmental status of each sub-bundle of the human SLF in neonates remain poorly understood. Here, we compared the morphological and microstructural characteristics of each branch of the SLF at two ages using diffusion MRI data from 40 healthy neonates and 40 adults. A multi-shell multi-tissue constrained spherical deconvolution (MSMT-CSD) algorithm was used to ensure the successful separation of the three SLF branches (SLF I, SLF II and SLF III). Then, between-group differences in the diffusion tensor imaging (DTI) and neurite orientation dispersion and density imaging (NODDI) metrics were investigated in all the SLF branches. Meanwhile, Mahalanobis distances based on all the diffusion metrics were computed to quantify the maturation of neonatal SLF branches, considering the adult brain as the reference. The SLF branches, excluding SLF II, had similar fibre morphology and connectivity between the neonatal and adult groups. The Mahalanobis distance values further supported the notion of heterogeneous maturation among SLF branches. The greatest Mahalanobis distance was observed in SLF II, possibly indicating that it was the least mature. Our findings provide a new anatomical basis for the early diagnosis and treatment of diseases caused by abnormal neonatal SLF development.

**Supplementary Information:**

The online version contains supplementary material available at 10.1007/s00429-022-02565-z.

## Introduction

The superior longitudinal fasciculus (SLF) is thought to be a long association fibre tract connecting the frontal lobe with the parietal lobe and the temporoparietal conjunction area. Based on its distinct anatomical connections in the cortical areas, the SLF can be divided into three subcomponents (SLF I, II and III) (Catani and Mesulam 2008; Catani and Thiebaut de Schotten 2012). These distinct sub-bundles have been linked to different cognitive functions, such as language, attention and visuospatial processing (Thiebaut De Schotten et al. 2011; Parlatini et al. 2017; Nakajima et al. 2020; Schurr et al. 2020). Previous studies have suggested that microstructural alterations in SLF sub-bundles are related to some neurodevelopmental and cognitive disorders. For example, greater fractional anisotropy (FA) of the right SLF II has been observed in the autism spectrum disorder (ASD) group (Fitzgerald et al. 2018), and higher axial diffusivity (AD) of the SLF I has been observed in individuals with attention deficit/hyperactivity disorder (ADHD) (Chiang et al. 2020). Hence, a segmentation study of the SLF and the morphological and microstructural exploration of each sub-bundle using diffusion MRI (dMRI) may be helpful for detecting cryptic white matter (WM) injury in neuropsychiatric disorders.

Many advanced tractography methods have been applied in in vivo studies of human adults and have enabled the separation and visualization of the three SLF branches (Makris et al. 2005; Thiebaut De Schotten et al. 2011; Schurr et al. 2020). The most dorsal component of the SLF (SLF I) connects the superior parietal lobule and precuneus (Brodmann areas, BA 5 and 7) with the superior frontal gyrus and some anterior cingulate areas (BA 8, 9 and 32). The middle pathway (SLF II) derived from the anterior intermediate parietal sulcus and the angular gyrus (BA 39) terminates within the middle part of the middle frontal gyrus (dorsolateral prefrontal cortex; BA 46/9). The most ventral branch (SLF III) originates from the supramarginal gyrus (BA 40) and ends in the inferior frontal gyrus (BA 44 and 45) (Yagmurlu et al. 2016). These in vivo findings correspond well with post-mortem blunt dissection results in the human brain (Thiebaut De Schotten et al. 2011; Wang et al. 2016). Earlier tracer studies in the rhesus monkey also demonstrated that the SLF is a system that consists of these three subcomponents (Petrides and Pandya 1984; Schmahmann and Pandya 2006). Further comparative studies have investigated the similarities and differences between the SLF connections in nonhuman primates and those in humans (Hecht et al. 2015).

To date, however, there have been very limited studies on the segmentation of SLF sub-bundles in human foetuses and neonates. Previous histological studies indicate that the outgrowth and ingrowth of long corticocortical pathways finish in the cortical plate during the late preterm phase (33–35 weeks post-conception) (Kostovic 2006). Another recent study found that at 32 to 35 gestational weeks (GW), the SLF can be divided into a deep, direct component (arcuate fasciculus, AF), and a more superficial indirect component using the WM dissection technique (Horgos et al. 2020). Nevertheless, the SLF is not prominently visible with diffusion tensor imaging (DTI) tractography even at birth (Zhang et al. 2007), and most neurodevelopmental studies have considered the entire SLF as a single entity (Ouyang et al. 2015, 2019; Yu et al. 2020). Obviously, SLF tracts estimated by DTI at a very early age do not reflect actual anatomical connectivity. There are some complicated factors contributing to this. First, the quality of dMRI images of foetuses and neonates is limited by the scanning time, magnetic field intensity and other conditions (Dubois et al. 2021). On the other hand, the SLF is one of the slowest maturing WM tracts with lower myelination, and many fibres intersect with it in its running areas (Zhang et al. 2007; Dubois et al. 2009; Geng et al. 2012). Previous studies have shown that the DTI technique may obtain more false negatives at points of crossing, kissing and twisting fibres, which can affect the ability to reconstruct streamlines of the SLF; thus, the full extent of the SLF was not identified (Makris et al. 2005; Kamali et al. 2014; Hecht et al. 2015). All these factors could lead to a reduction in DTI tracking accuracy and loss of structural properties of SLF branches. Constrained spherical deconvolution (CSD) can overcome the crossing fibre limitations inherent in the diffusion tensor model and minimize the bias by tracking through regions with multiple fibre orientations (Thiebaut De Schotten et al. 2011; Jeurissen et al. 2014; Wang et al. 2016; Nakajima et al. 2020; Amemiya et al. 2021), obtaining more reliable tractography results of the neonatal SLF branches.

Additionally, when measuring development, DTI parameters suffer from partial volume effects owing to free-water contamination and complex fibre orientations in the immature brain (Lynch et al. 2020). Thus, to investigate the development status of SLF sub-bundles from a more precise microstructural perspective, some new technologies should be applied to analyse high-quality diffusion images during the foetal and neonatal periods. Neurite orientation dispersion and density imaging (NODDI) is a multi-compartment model that can overcome these limitations, and it has high specificity to characterize the microstructural features of WM development. Specifically, in WM, NODDI can differentiate three tissue types in a voxel: (1) the intra-neurite compartment, referring to the space bounded by the membrane of axons that enables quantitative measures of the packing density and myelination of axons, termed the neurite density index (NDI), and the orientation coherence of neurites, termed the orientation dispersion index (ODI); (2) the extra-neurite compartment, referring to the space around the axons, which is occupied by oligodendrocytes; and (3) the free-water compartment, mainly representing cerebrospinal fluid (CSF) (Zhang et al. 2012; Lynch et al. 2020). The NODDI microstructural parameters may provide unique insight into the complex development of WM tracts and provide complementary information to that provided with DTI.

According to the above considerations, we used multi-shell dMRI data from the Developing Human Connectome Project (dHCP) and the Human Connectome Project (HCP) to reconstruct the trajectories of the three SLF subcomponents during the neonatal and young adult periods via the multi-shell multi-tissue CSD (MSMT-CSD) method. Then, we investigated the microstructural differences in the SLF sub-bundles between the two groups by measuring DTI and NODDI metrics. Finally, and of particular interest, to quantify the maturational degree of each SLF branch in the neonatal group, Mahalanobis distances based on the combination of all the DTI and NODDI parameters were computed, considering the adult brain as the reference.

## Materials and methods

### Study sample

High-resolution T2-weighted and dMRI data for 40 normal term-born neonates were acquired from the first data release of the dHCP (http://www.developingconnectome.org). These babies were scanned at 37–44 weeks post-menstrual age (PMA). The research of the dHCP was approved by the UK Health Research Authority (Research Ethics Committee reference number: 14/LO/1169).

We chose 40 healthy young adults (ages 22–35 years, 24 males) with high-quality T2-weighted and dMRI data from the HCP (https://www.humanconnectome.org) of the Washington University–University of Minnesota (WU-Minn) Consortium. The WU-Minn HCP Consortium obtained full informed consent from all participants, and the research procedures and ethical guidelines were followed in accordance with Washington University institutional review board approval (Mapping the Human Connectome: Structure, Function and Heritability; IRB # 201,204,036).

The demographic information of the 40 neonates and 40 young adults is shown in Table 1. These subjects were all visually inspected by an experienced anatomist, and no evidence of local lesions on conventional structural images was detected.

**Table 1:** Demographic characteristics of the study sample

Group	Neonatal group (*n* = 40)	Adult group (*n* = 40)
GA at birth, mean ± SD	38.99 ± 1.66 (weeks)	–
Age at scan, mean ± SD	39.89 ± 2.08 (weeks, PMA)	28.8 ± 3.91 (years)
Male, *n* (%)	25 (62.5%)	24 (60%)

*GA* gestational age; *PMA* post-menstrual age

### MRI acquisition

All scans from the dHCP were conducted on a 3.0 T Philips Achieva MRI scanner equipped with a dedicated neonatal brain imaging system, which has specially designed immobilization devices that conform to the shape of the infants’ head and assists in keeping the infants asleep and minimizing gross head motion (Hughes et al. 2017). Diffusion MRI data were acquired with a monopolar spin echo echo-planar imaging (SE-EPI) Stejskal-Tanner sequence (TR/TE, 3800/90 ms; field of view (FOV), 150 × 150 × 96 mm^3^; matrix, 128 × 128 × 64). The acquisition time was shorter than 20 min. There were 4 different *b*-value shells (0, 400, 1000 and 2600 s/mm^2^) and 300 diffusion encoding orientations (20, 64, 88 and 128 per *b*-value shell) for each subject (Hutter et al. 2018). T2-weighted scans were acquired using a turbo spin echo (TSE) sequence (TR/TE, 12 s/156 ms; resolution (mm) 0.8 × 0.8 × 1.6).

All the HCP subjects were scanned on a 3.0 T Siemens connectome—Skyra scanner with a customized protocol. For each subject, the multi-shell dMRI data have an isotropic spatial resolution of 1.25 mm and over 270 gradient directions distributed over three *b* values (1000, 2000, 3000 s/mm^2^). T1-weighted images were acquired using the 3D MPRAGE sequence with 0.7 mm isotropic resolution (Sotiropoulos et al. 2013).

### Diffusion data preprocessing

A fully automated pipeline was used to preprocess the dHCP neonatal diffusion data (Bastiani et al. 2019). Denoising and Gibbs-ringing artefact removal were performed first as part of the preprocessing (Kellner et al. 2016; Veraart et al. 2016). The next FSL EDDY and FSL TOPUP procedures comprised of correcting for subject motion artefacts and susceptibility distortions (Andersson et al. 2003). Similarly, the data for the adults underwent distortion correction via the HCP preprocessing pipeline. More details on the data preprocessing method were previously reported (Glasser et al. 2013; Bastiani et al. 2019).

### FOD computation and global tractography

First, 3-tissue response functions representing single-fibre WM, grey matter (GM) and CSF were estimated from the data themselves using a robust and fully automated unsupervised method (Dhollander and Connelly 2016). Next, MSMT-CSD was performed to obtain WM-like fibre orientation distributions (WM-FODs) (Supplementary Fig. 1) as well as GM-like and CSF-like compartments in all voxels (Tournier et al. 2007, 2019). Then, we performed global tractography on the basis of obtaining an accurate representation of WM-FODs using a deterministic algorithm, namely, SD_STREAM as implemented in MRtrix (https://www.mrtrix.org/) (Tournier et al. 2012). Most tractography parameters were set to the default values, except for the following parameters: the maximum angle between successive steps was 45°, the FOD amplitude cut-off was 0.06, and the desired number of streamlines to be selected was 10 million.

### ROI-based segmentation of the SLF

**Fig. 1:**
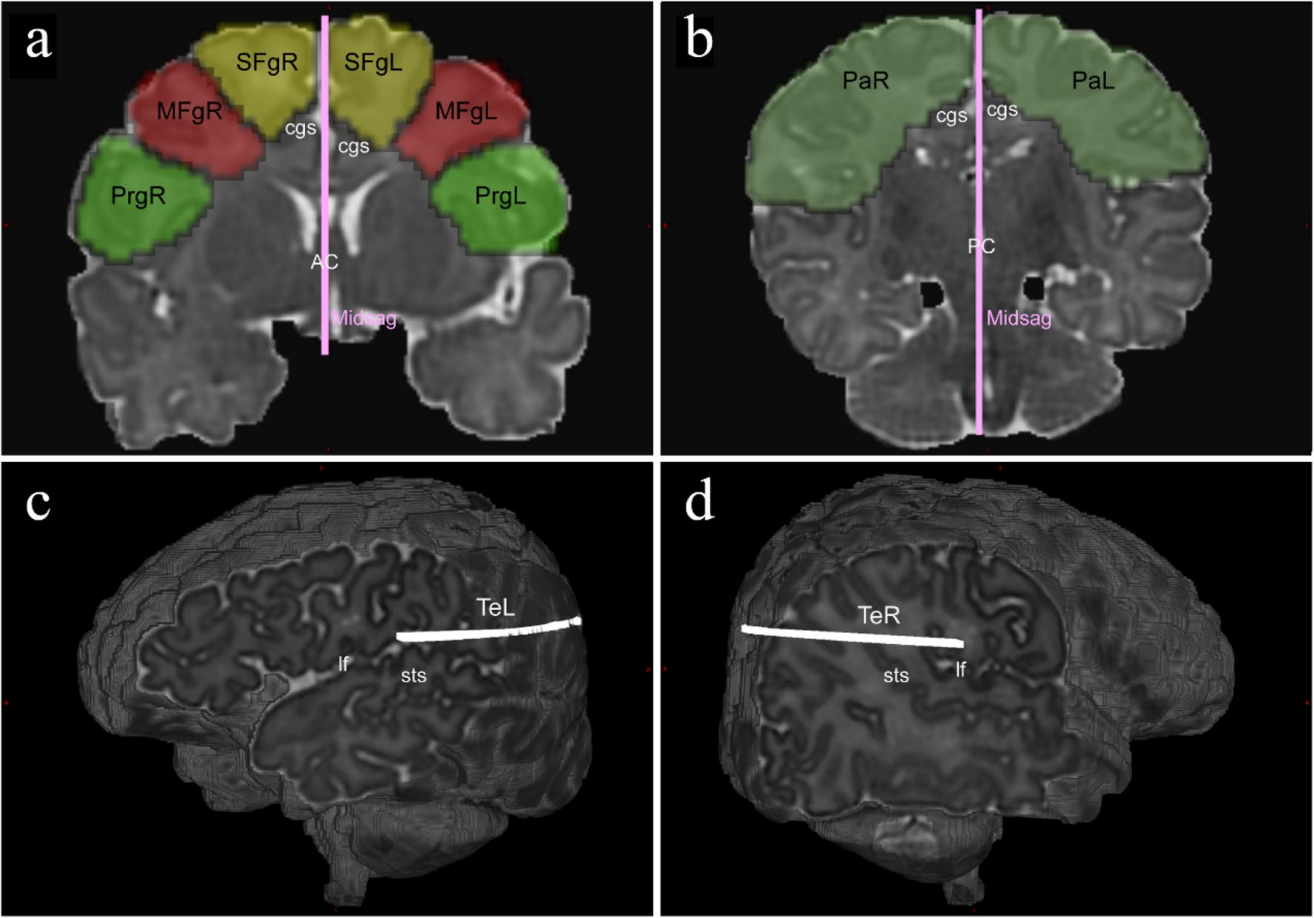
Delineation of inclusion and exclusion ROIs used for segmentation of left and right SLF three branches, overlaid on a synthetic T2-weighted image in a representative participant of neonatal group. **a** Coronal ROIs in the left and right frontal lobes. **b** Coronal ROIs in the left and right parietal lobes. **c–d** Axial ROIs in the left and right hemispheres used to exclude the streamlines of arcuate fasciculus projecting to the temporal lobe. *SFgL* superior frontal gyrus left, *SFgR* superior frontal gyrus right, *MFgL* middle frontal gyrus left, *MFgR* middle frontal gyrus right, *PrgL* precentral gyrus left, *PrgR* precentral gyrus right, *PaL* parietal left, *PaR* parietal right, *TeL* temporal left, *TeR* temporal right, *AC* anterior commissure, *PC* posterior commissure, *Midsag* mid-sagittal plane, *cgs* cingulate sulcus, *sts* superior temporal sulcus, *lf* lateral fissure

**Fig. 2:**
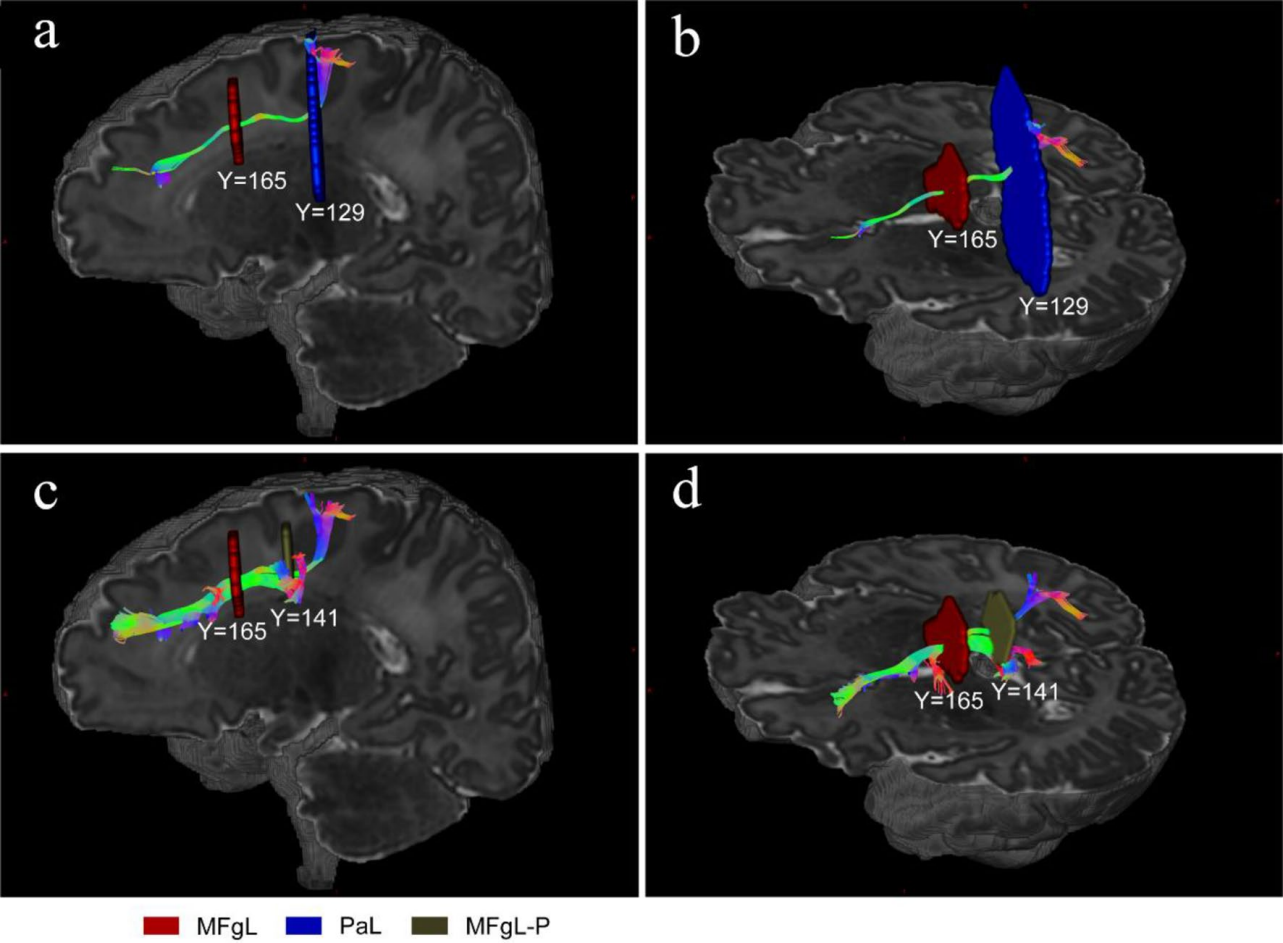
The fiber tracking of left SLF II using different combinations of ROIs in a neonatal brain. **a–b** A sagittal view of the fiber tracking of left SLF II using the “MFgL” AND “PaL” ROIs. **c–d** A sagittal view of the fiber tracking of left SLF II using the “MFgL” AND “MFgL-P” ROIs. *MFgL* middle frontal gyrus left, *PaL* parietal left, *MFgL-P* middle frontal gyrus left-posterior

The regions of interest (ROIs) were manually drawn on the FOD image in native diffusion space for each participant independently using ITK-SNAP (www.itksnap.org/) software (Yushkevich et al. 2006), and the SLF was segmented into three parts: SLF I, SLF II and SLF III from the results of global tractography. In general, our protocol of delineating ROIs for isolating the three components of the SLF was mainly inspired by previous work (Thiebaut De Schotten et al. 2011, 2012; Hecht et al. 2015; Amemiya et al. 2021). Figure 1 depicts the positions of waypoint ROIs used for segmentation of SLF, overlaid on a synthetic T2-weighted image in a representative participant of the neonatal group. These labelled ROIs can be split into two categories: one was delineated around the WM of the superior, middle and inferior/precentral frontal gyri in an anterior coronal section including the anterior commissure (Fig. 1a), and another coronal “AND” ROIs covered the WM of the parietal lobe superior to the lateral fissure in a posterior coronal section including the posterior commissure (Fig. 1b). The former ROIs served to classify SLF into SLF I, SLF II and SLF III (yellow, red and green; Fig. 1a). The latter ROIs were used to constrain the trajectories of fibre bundles (Fig. 1b). Besides, we delineated several “NOT” ROIs to exclude the hybrid fibre bundles adjacent to or crossing the SLF. For example, the callosal fibres were excluded using a “NOT” ROI in the mid-sagittal plane (pink; Fig. 1a, b), while a “NOT” ROI in the axial slice covering the temporal lobe was used to exclude the streamlines of arcuate fasciculus projecting to the temporal lobe (white; Fig. 1c, d). Supplementary Fig. 2 depicts the positions of waypoint ROIs used for segmentation of the SLF, overlaid on a synthetic T1-weighted image in a representative participant of the adult group. Table 2 provides the different combinations of ROIs for the tractography of distinct SLF branches. Notably, we adopted a different tracking protocol for SLF II in the neonatal group. Figure 2 shows that we used the “MFgL-P” ROI (Fig. 2c, d) that is behind the “anterior commissure” and ahead of the “posterior commissure” instead of the “PaL” ROI (Fig. 2a, b) to obtain a better tracking result of SLF II streamlines in the neonatal group. Some fibres belonging to SLF II that did not reach the “PaL” ROI were saved.

**Table 2:** ROIs combinations for the tractography of distinct SLF branches

	Adults	Neonates
Left SLF I	PaL ‘AND’ SFgL ‘NOT’ TeL ‘NOT’ Midsag	PaL ‘AND’ SFgL ‘NOT’ TeL ‘NOT’ Midsag
Left SLF II	PaL ‘AND’ MFgL ‘NOT’ TeL ‘NOT’ Midsag	MFgL-P ‘AND’ MFgL ‘NOT’ TeL ‘NOT’ Midsag
Left SLF III	PaL ‘AND’ PrgL ‘NOT’ TeL ‘NOT’ Midsag	PaL ‘AND’ PrgL ‘NOT’ TeL ‘NOT’ Midsag
Right SLF I	PaR ‘AND’ SFgR ‘NOT’ TeR ‘NOT’ Midsag	PaR ‘AND’ SFgR ‘NOT’ TeR ‘NOT’ Midsag
Right SLF II	PaR ‘AND’ MFgR ‘NOT’ TeR ‘NOT’ Midsag	MFgR-P ‘AND’ MFgR ‘NOT’ TeR ‘NOT’ Midsag
Right SLF III	PaR ‘AND’ PrgR ‘NOT’ TeR ‘NOT’ Midsag	PaR ‘AND’ PrgR ‘NOT’ TeR ‘NOT’ Midsag

*SFgL* superior frontal gyrus left, *SFgR* superior frontal gyrus right, *MFgL* middle frontal gyrus left, *MFgR* middle frontal gyrus right, *PrgL* precentral gyrus left, *PrgR* precentral gyrus right, *PaL* parietal left, *PaR* parietal right, *TeL* temporal left, *TeR* temporal right, Midsag mid-sagittal plane, *MFgL*-*P* middle frontal gyrus left-posterior, *MFgR*-*P* middle frontal gyrus right-posterior

### Defining the cortical ROIs based on the AAL template

To determine the cortical ROIs connected by the three branches of the SLF, we registered the neonatal atlases published by Shen et al. (Shi et al. 2011) to the individual diffusion space of the 40 newborns using Advanced Normalization Tools (ANTs, http://stnava.github.io/ANTs/) software and divided each newborn individual's brain into 90 anatomical regions (Supplementary Fig. 3). T2 images of each newborn individual were also registered into the corresponding diffusion space to display the tractography results. The same procedure was used for the adult group. We also divided each adult brain into 90 anatomical regions based on the adult AAL atlas (Tzourio-Mazoyer et al. 2002).

### Measurements of DTI and NODDI parameters

“*b* = 0 and *b* = 1000 s/mm^2^” data were extracted using MRtrix to calculate the DTI metrics (fractional anisotropy [FA], mean diffusivity [MD], axial diffusivity [AD] and radial diffusivity [RD]) using FSL software (https://fsl.fmrib.ox.ac.uk/fsl). For NODDI model fitting, we calculated the main NODDI metrics (NDI, ODI) through the NODDI MATLAB Toolbox software (https://www.nitrc.org/projects/noddi_toolbox) using all four shells data (Zhang et al. 2012) (Supplementary Fig. 4).

**Fig. 3:**
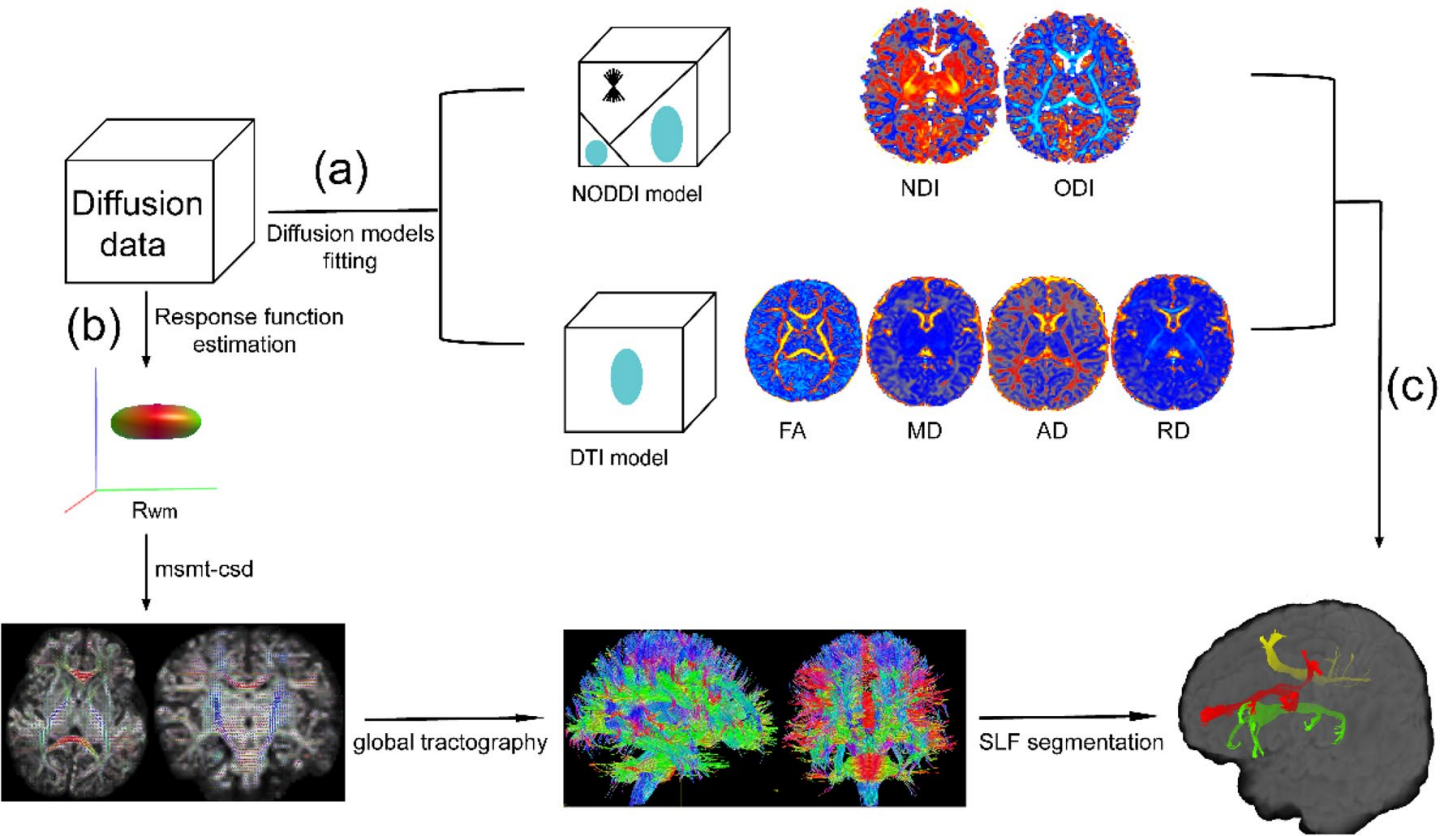
Outline of the analysis pipeline. **a** The process of diffusion models fitting. **b** The segmentation process of SLF three branches. **c** Acquisition of mean metric values of each SLF branch

Combined with the results of fibre tracking as well as DTI and NODDI parameter values, we used the “tcksample” command in MRtrix to obtain the DTI and NODDI parameter values of sampling points in each SLF branch. Finally, we used an in-house Python script to compute the mean metric value over the tract by averaging the measurements from all sampling points of all fibres, which can be downloaded from https://data.mendeley.com/datasets/5yymds8v5b/1. The same analysis pipeline was performed for both the neonatal and adult groups (Fig. 3).

### Calculation of the Mahalanobis distances

To quantify the maturational degree of each SLF branch in the neonatal brain, complex Mahalanobis distances (M) based on the combination of all the NODDI and DTI parameters were calculated between a neonate and a reference group of adults by using the following equations:

1 $$M^{2} \left( {\vec{x}} \right) = \left( {\vec{x} - \vec{\mu }} \right)^{T} \sum\nolimits^{ - 1} \left( {\vec{x} - \vec{\mu }} \right) = \left( {\vec{x} - \vec{\mu }} \right)^{T} \left( {V^{T} \cdot \lambda \cdot V} \right)^{ - 1} \left( {\vec{x} - \vec{\mu }} \right)$$

2 $$M^{2} \left( {\vec{x}} \right) = \sum\nolimits_{i}^{n} {\left( {\left( {\vec{x} - \vec{\mu }} \right)\vec{v}_{i} } \right)^{2}/\lambda_{i} }$$

In the above formula (1), $\overrightarrow{x}$ is a multivariate vector describing a neonatal SLF sub-bundle and $\overrightarrow{\mu }$ and *∑* are the mean vector and covariation matrix for parameters of the corresponding bundle in the adult group, respectively. The smaller this Mahalanobis distance, the closer the SLF branch of neonate to its mature adult stage (Kulikova et al. 2015). The matrix *∑* could be viewed as the product of two matrices ($\mathrm{V}$ and $\lambda$), one for rotation and one for scaling, and so can its inverse. The latter matrix $\lambda$ comprised of the eigenvalues of the covariation matrix *∑* and was used to scale the variables so that the variance for each diffusion metric equals 1 (Brereton 2015). In other words, the different metrics were scaled to be all at the same scale by the corresponding eigenvalues of covariance matrix in the process of Mahalanobis distance calculation. Therefore, the Mahalanobis distance is scale invariant, meaning that the different order of magnitude of the diverse metrics do not bias the analysis (Bedrick et al. 2000; Flores-Guerrero et al. 2021). Mahalanobis distance can be equally calculated using the formula (2), where ${\overrightarrow{v}}_{i}$ and ${\lambda }_{i}$ are the n eigenvectors and eigenvalues of the covariation matrix *∑* (Kulikova et al. 2015). In this work, Mahalanobis distances were calculated by using the combination of NODDI and DTI metrics (NDI, ODI, FA, MD, AD, RD), named M_DT-NODDI_, which considered the multiple metrics together and provided more microstructural information for assessing white matter maturation than that based on just DTI or NODDI metrics (Li et al. 2022).

## Statistical analysis

SPSS software, version 22.0 (IBM, Armonk, New York) was used for statistical analysis. In our analysis, we tested the normality of distributions using the ‘Shapiro–Wilk test’ before further statistical detection, and we used standard parametric statistics to draw statistical inferences only when a Gaussian distribution was confirmed for each dependent variable.

Analyses of asymmetries between left and right hemispheres were quantified by a lateralization index (LI), which was calculated for the DTI and NODDI measures of the three branches of the SLF according to the following formula: Lateralization index = (Left–Right)/(Left + Right). Positive values indicate left lateralization, whereas negative values indicate right lateralization. A one-sample *t* test (test value = 0) was used to assess the lateralization of the SLF I, II and III.

In addition, we performed an intragroup Pearson's correlation analysis with scan age for all investigated metrics of each SLF branch. To correct for the number of SLF branches, Bonferroni–Dunn method was also provided to determine the significance of correlation analysis at *p* = 0.008, which is equivalent to *p* = 0.05 after Bonferroni correction for six correlation analyses. Then, we compared the group differences in all DTI and NODDI parameters between neonates and adults using two-sided Student's *t* test. Statistical significance was also determined using the Bonferroni correction, with *p* = 0.008 (0.05/6). When we averaged the left and right metrics of the same SLF branch, all the threshold for statistical significance was set to *p* = 0.01 (0.05/3). In addition, the magnitude of between-group differences in metrics was quantified using Cohen's d (Grinberg et al. 2017). We took the parameter as the subscript of Cohen's d to represent the effect value of the calculated parameter; for example, d_FA_ is Cohen's d for FA, d_NDI_ is Cohen's d for NDI, and so on.

Finally, the non-parametric Wilcoxon signed rank test was performed to compare the Mahalanobis distances between SLF branches of neonates. Statistical significance was determined using the Bonferroni correction, with *p* = 0.01 (0.05/3). Correlations between Mahalanobis distances of SLF branches and PMA were performed by using the non-parametric Spearman's rank correlation.

## Results

### Comparisons of trajectories of SLF branches between the neonatal and adult groups

**Fig. 4:**
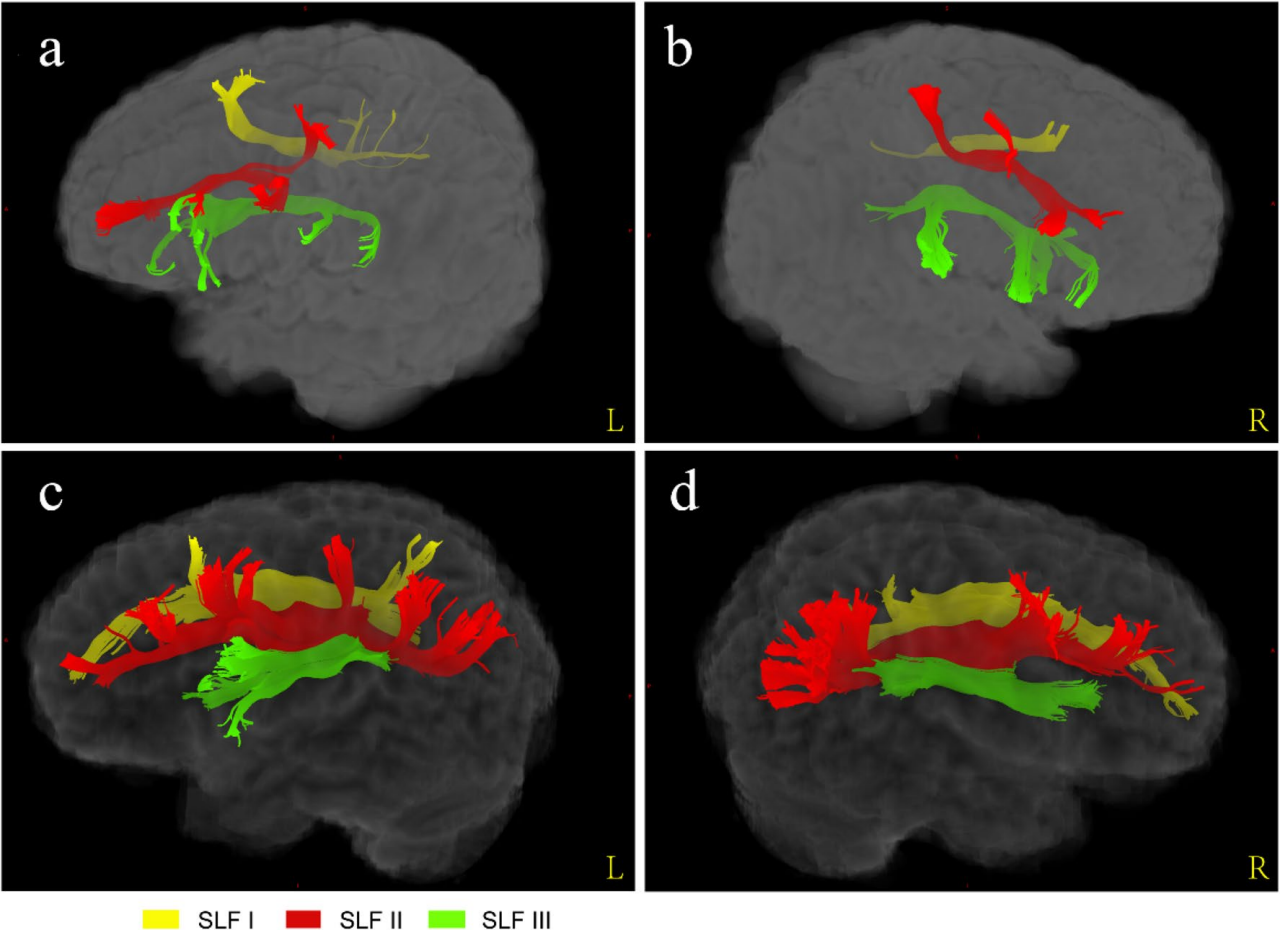
A three-dimensional view of SLF three branches. **a–b** Three SLF branches in the left and right hemisphere view from a representative participant of neonatal group. **c–d** Three SLF branches in the left and right hemisphere view from a representative participant of adult group

We conducted whole-brain fibre tracking for the 40 neonates as well as 40 young adults and then obtained three SLF segments (Fig. 4) by manually delineating ROIs on the WM-FOD map for each participant independently.

**Fig. 5:**
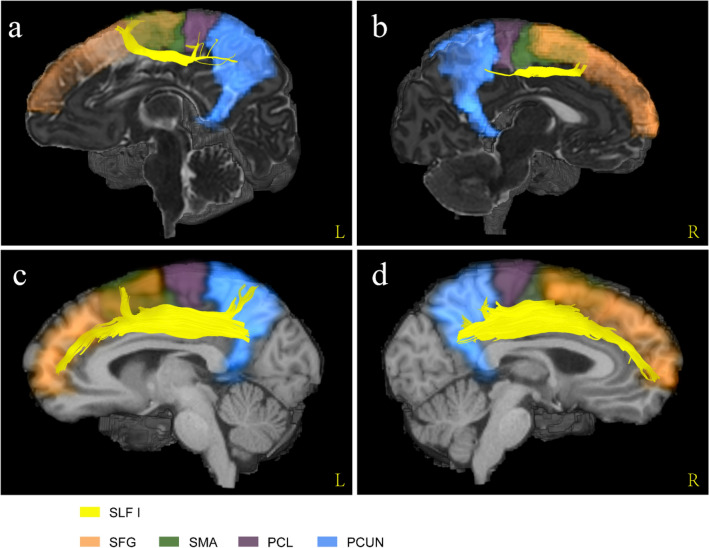
The comparison of cortical regions that SLF I connected between neonatal and adult groups. **a–b** A sagittal view of left and right SLF I from a representative participant of neonatal group. **c–d** A sagittal view of left and right SLF I from a representative participant of adult group. *SFG* superior frontal gyrus, *SMA* supplementary motor area, *PCL* paracentral lobule, *PCUN* precuneus

**Fig. 6:**
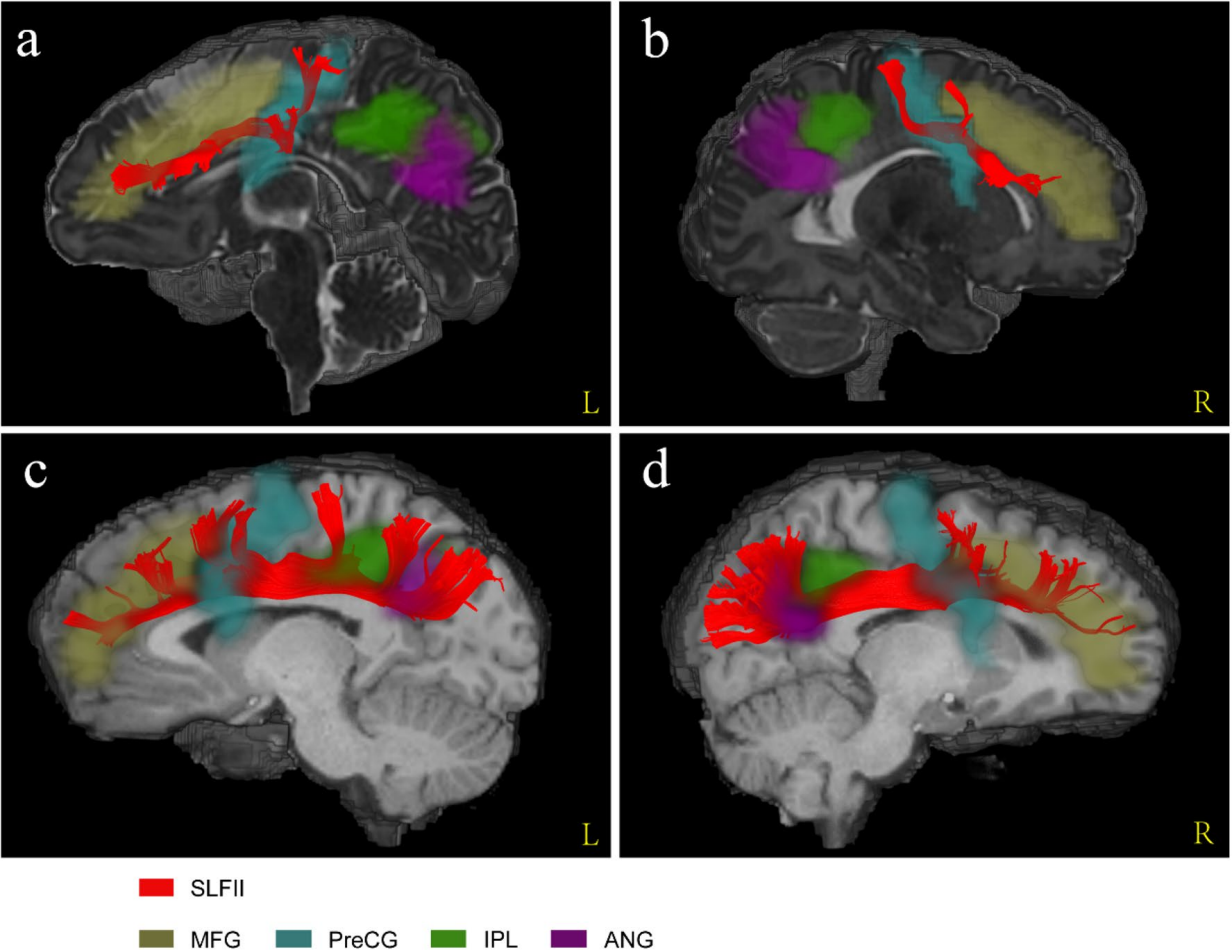
The comparison of cortical regions that SLF II connected between neonatal and adult groups. **a–b** A sagittal view of left and right SLF II from a representative participant of neonatal group. **c–d** A sagittal view of left and right SLF II from a representative participant of adult group. *MFG* middle frontal gyrus, *PreCG* precentral gyrus, *IPL* inferior parietal lobule, *ANG* angular gyrus

**Fig. 7:**
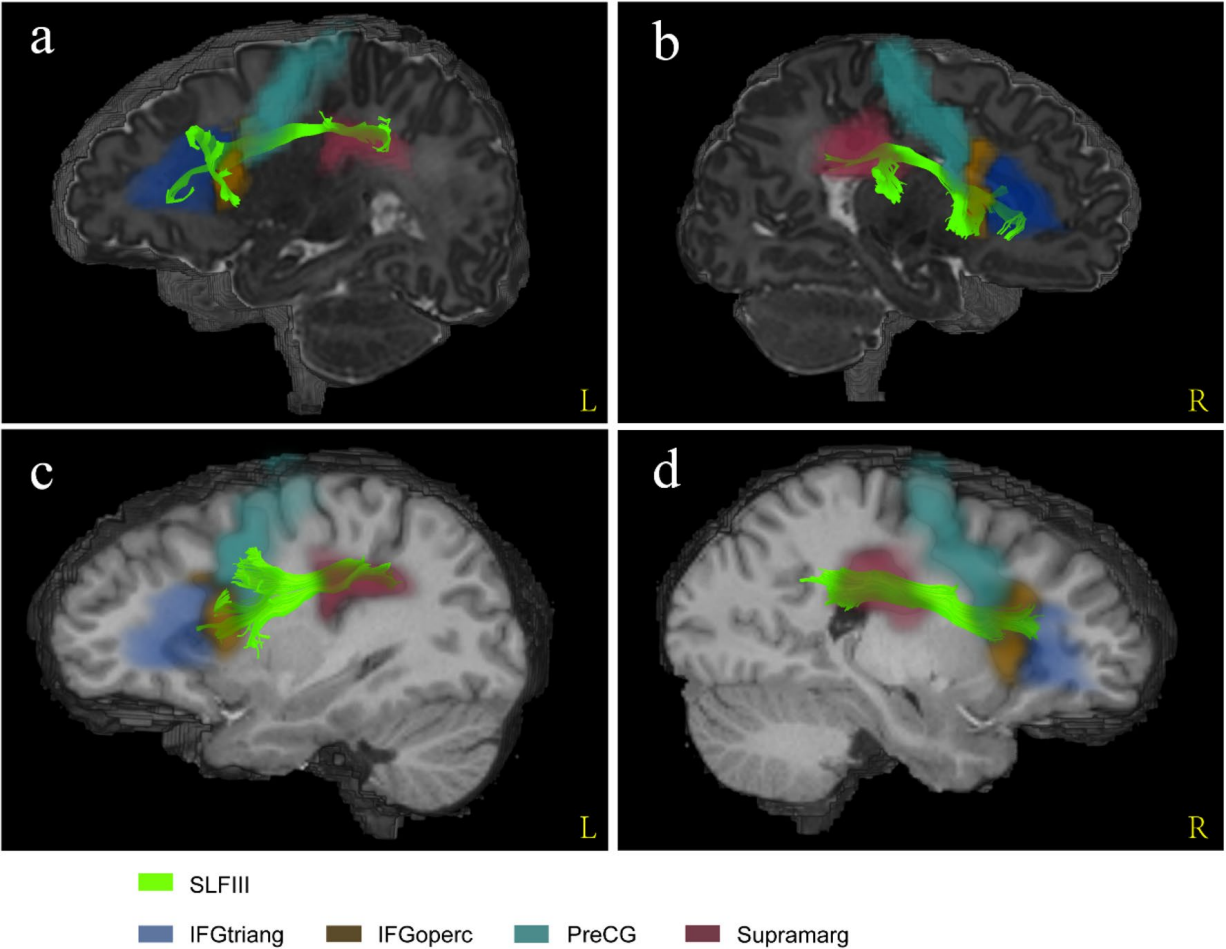
The comparison of cortical regions that SLF III connected between neonatal and adult groups. **a–b** A sagittal view of left and right SLF III from a representative participant of neonatal group. **c–d** A sagittal view of left and right SLF III from a representative participant of adult group. IFGtriang inferior frontal gyrus (triangularis), IFGoperc inferior frontal gyrus (opercularis), *PreCG* precentral gyrus, *SMG* supramarginal gyrus

Combined with the registered AAL atlases, we found that the fibre morphology and connected cortical regions of each SLF branch in the neonates were similar to those in the adults. Figure 5 shows that SLF I connected the superior frontal gyrus, supplementary motor area, paracentral lobule and precuneus in both the neonatal and adult groups. SLF II was located more laterally and inferiorly than SLF I (Fig. 4). In the adult group, SLF II originated from the angular gyrus to the middle frontal gyrus and precentral gyrus (Fig. 6c, d). Notably, the streamlines of SLF II did not reach the parietal areas, such as the inferior parietal lobule and angular gyrus, due to the lower maturation in the neonatal group (Fig. 6a, b). SLF III was located on the ventral side of SLF II (Fig. 4). In the sagittal planes where SLF II and SLF III existed simultaneously, the fibre bundles running with a more dorsal direction were regarded as SLF II, while the remaining fibres with a more ventral direction were regarded as SLF III. As shown in Fig. 7, the cortical regions that SLF III connected in the neonatal group were also similar to those in the adult group, originating from the supramarginal gyrus and precentral gyrus and terminating in the inferior frontal gyrus (pars triangularis and pars opercularis).

### Microstructural changes in SLF branches with increasing age at scan

The statistical analyses of LI values did not reveal any inter-hemispheric differences in the SLF branches of neonates (Supplementary Fig. 5). Therefore, we averaged the left and right metrics of the same branch for the following analyses. As shown in Table 3, for the neonatal group, NDI and FA strongly increased over 37–44 weeks PMA, following a linear growth pattern for all branches of the SLF. MD and RD were negatively associated with PMA. All age-related changes were still statistically significant after Bonferroni–Dunn correction. Remarkably, however, AD and ODI showed no significant relationship with increasing PMA for all the subcomponents after Bonferroni correction. The scatter plots of NODDI and DTI metrics varying with PMA in the neonatal SLF branches can be seen in Supplementary Figs. 6–11. The detailed statistical results are reported in Supplementary Table 1. In contrast to neonates, adults showed no obvious microstructural changes during the period of 22–35 years.

**Table 3:** Pearson’s correlation coefficient (*r*) values and corresponding *p* values between diffusion metrics and post-menstrual age in various SLF branches among neonates

		SLF I	SLF II	SLF III
NDI	*r*	0.5633	0.5568	0.5496
	*p*	0.0002^**^	0.0002^**^	0.0002^**^
	*t*	4.2026	4.1321	4.0554
	df	38	38	38
ODI	*r*	– 0.2018	– 0.1525	– 0.01245
	*p*	0.2118	0.3475	0.9392
	*t*	– 1.2701	– 0.9512	–0.0768
	df	38	38	38
FA	*r*	0.5306	0.5628	0.4737
	*p*	0.0004^**^	0.0002^**^	0.002^*^
	*t*	3.8588	4.1971	3.3157
	df	38	38	38
MD	*r*	-0.4837	–0.4438	– 0.4993
	*p*	0.0016^*^	0.0041^*^	0.0010^**^
	*t*	– 3.4068	– 3.0529	– 3.5524
	df	38	38	38
AD	*R*	– 0.3150	–0.2228	–0.3753
	*P*	0.0477	0.1670	0.0170
	*T*	– 2.0460	– 1.4088	– 2.4960
	df	38	38	38
RD	*R*	– 0.5236	– 0.4902	– 0.5275
	*P*	0.0005^**^	0.0013^*^	0.0005^**^
	*T*	– 3.7885	– 3.4669	– 3.8276
	df	38	38	38

The significance of the correlation analysis after Bonferroni–Dunn correction was defined as *p < 0.01; **p ≤ 0.001

### Microstructural comparisons of SLF branches between the neonatal and adult groups

**Fig. 8:**
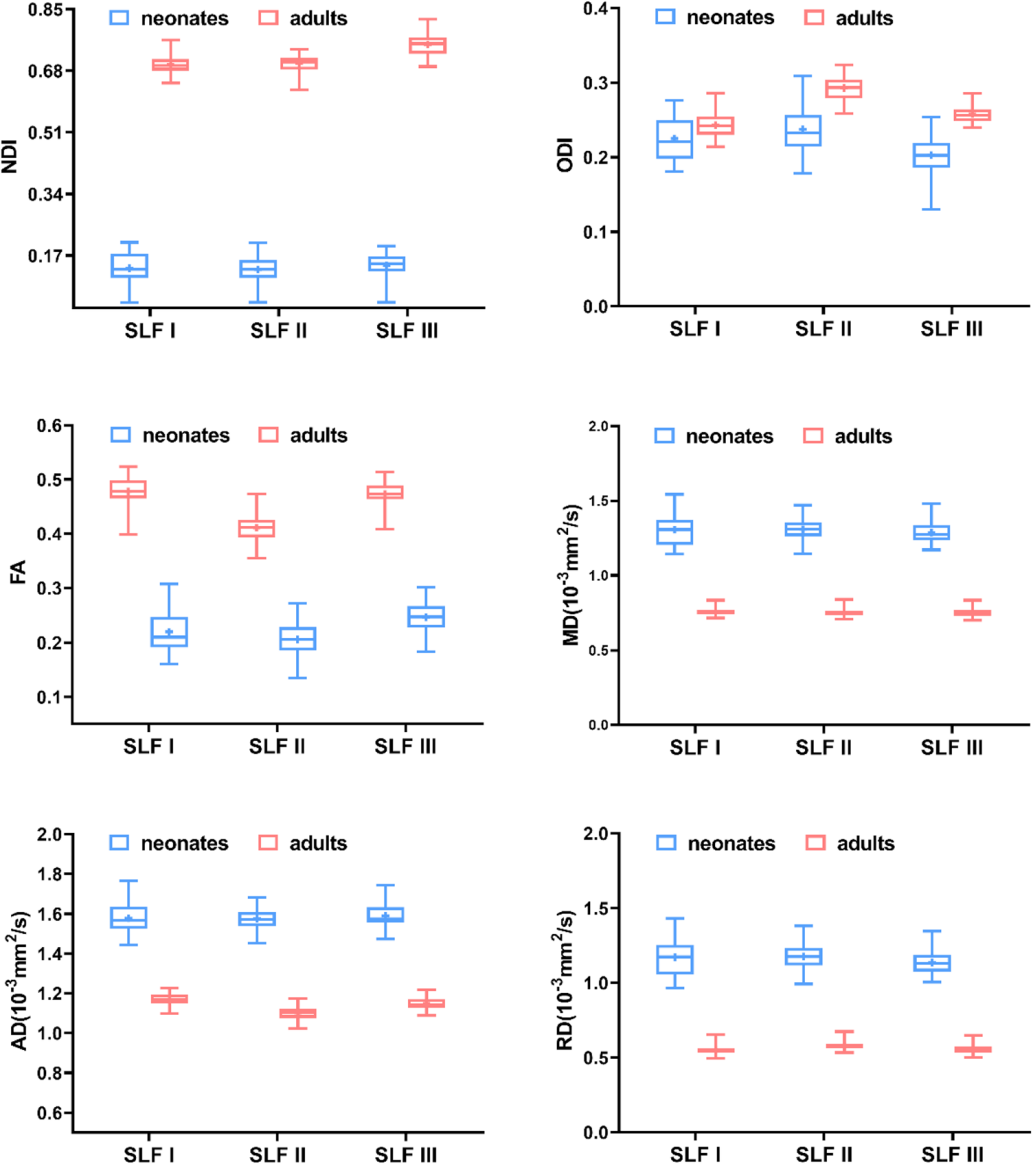
Quantification of NODDI and DTI parameters of SLF branches in neonates and adults. The differences in mean parameter values across the branches of the SLF between the neonatal and adult groups were all statistically significant after Bonferroni correction (*p* < 0.01)

We first observed the microstructural differences of SLF branches between the neonatal and adult groups based on the comparisons of the mean parameter values over each of the groups. The group mean values and corresponding standard deviations of DTI and NODDI parameters in the SLF branches for the neonatal and adult groups are reported in Supplementary Table 2a and 2b, respectively. As shown in Fig. 8, the between-group differences in SLF branches were very large, as demonstrated by the large differences in length between the red (adults) and blue (neonates) bars for the NODDI and DTI metrics except for the ODI. Our statistical results also showed that the NODDI and DTI parameter values significantly differed between the neonatal and adult groups (the adjusted *p* values after Bonferroni correction were all lower than 0.0001, except for the *p* values of the comparison results of the ODI for SLF I between the two groups, which were lower than 0.01), as illustrated in Table 4.

**Table 4:** Group differences in all NODDI and DTI parameters in various SLF branches between neonates and adults

		SLF I	SLF II	SLF III
NDI	*p*	< 0.000001	< 0.000001	< 0.000001
	t	69.54	83.20	81.10
	df	78	78	78
ODI	*p*	0.003681	< 0.000001	< 0.000001
	*t*	3.355	10.85	12.92
	df	78	78	78
FA	*p*	< 0.000001	< 0.000001	< 0.000001
	*t*	33.55	31.78	38.99
	df	78	78	78
MD	*p*	< 0.000001	< 0.000001	< 0.000001
	*t*	32.33	44.97	43.99
	df	78	78	78
AD	*p*	< 0.000001	< 0.000001	< 0.000001
	*t*	31.65	47.37	42.63
	df	78	78	78
RD	*p*	< 0.000001	< 0.000001	< 0.000001
	*t*	31.09	40.70	42.20
	df	78	78	78

The *p* values in the table have been corrected by the Bonferroni correction

**Fig. 9:**
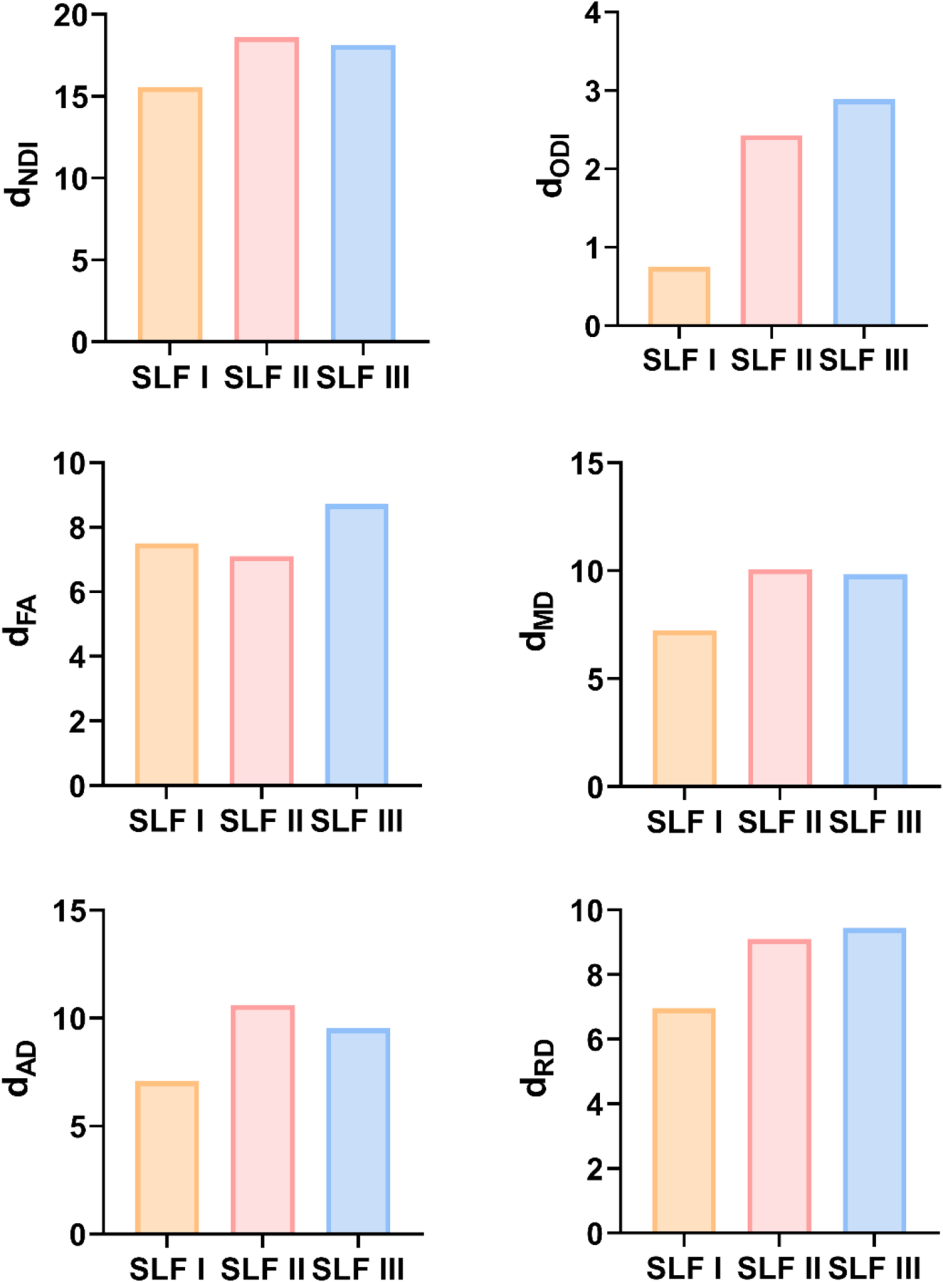
The values of Cohen’s d of NODDI and DTI metrics for the three SLF branches. The highest Cohen’s d is assumed to represent the greatest microstructural changes between neonates and adults, indicating the least mature state

In addition, we computed Cohen’s d effect size to quantify the magnitude of age-related metric changes between the two groups (Supplementary Table 3). Among these parameters, the smallest Cohen's d values were observed for the ODI of all the SLF branches. Figure 9 depicts the effect size of all the metrics for the investigated SLF branches. We found that the absolute Cohen's d values for the NODDI and DTI parameters of the SLF I were lower than those for SLF II and III, except for the d_FA_. Besides, SLF II exhibited the largest d_NDI_, d_MD_ and d_AD_ values among the three branches. The largest d_ODI_, d_FA_ and d_RD_ values were found for SLF III. These differences of Cohen’s d indicate that the maturity of SLF branches was not consistent.

**Fig. 10:**
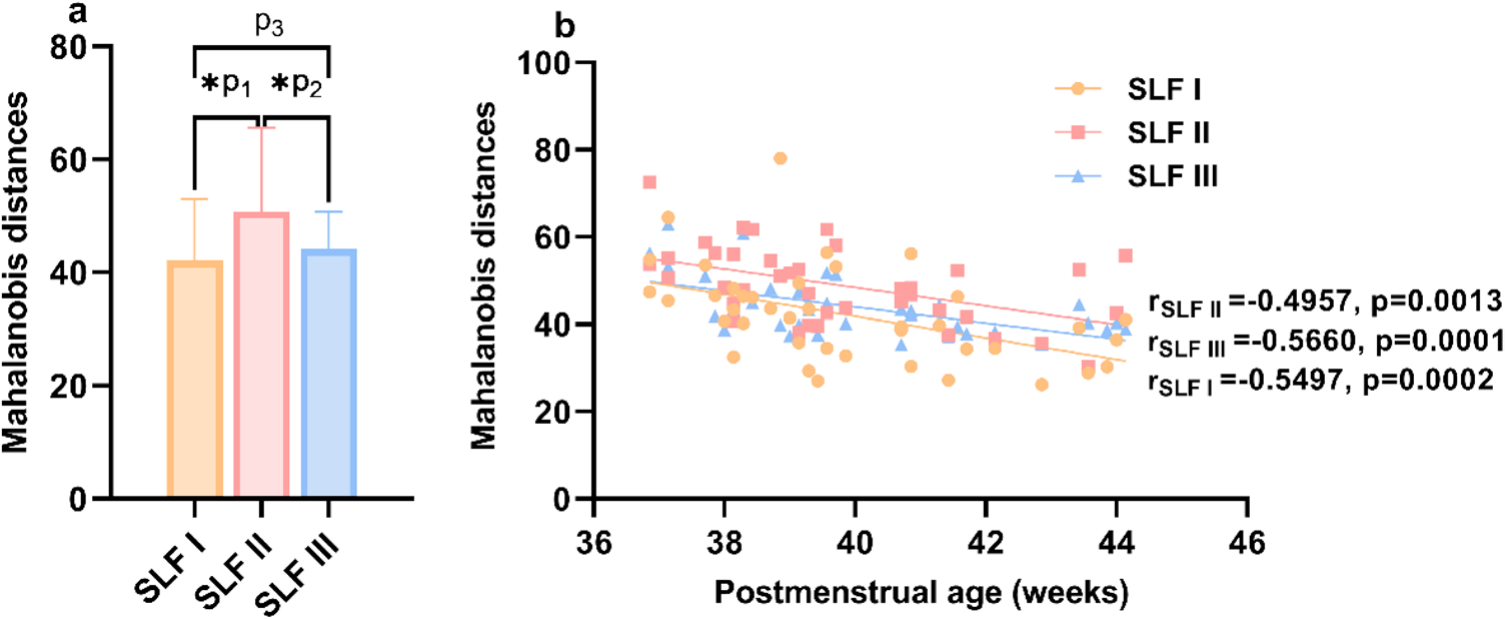
The maturation order of the various SLF branches as revealed by the Mahalanobis distance. **a** Mean Mahalanobis distances for the three SLF branches. SLF II has the highest Mahalanobis distance, meaning the least mature state (*The statistical significance remained when Bonferroni correction was set at *p* = 0.01 (0.05/3); *p_1_ < 0.0001, *p_2_ < 0.0001, *p*_3_ = 0.0208). **b** Correlation between Mahalanobis distances and post-menstrual age in neonates. The Mahalanobis distances decreased with the neonates’ age in all SLF branches and were fitted with linear models over this short developmental period

To validate the conclusions drawn from the qualitative comparisons of two Cohen’s *d* values, Mahalanobis distances were also calculated to provide statistical comparisons of the maturity of SLF branches, which allowed considering multiple metrics simultaneously. The results of Mahalanobis distances can be seen in Fig. 10. Statistical results indicate that SLF II has the highest Mahalanobis distance and the lowest maturity compared with SLF I and SLF III. There was no statistically significant difference in the Mahalanobis distance between SLF I and SLF III. In addition, the Mahalanobis distance decreased with PMA in all SLF branches of neonates, reflecting the increase in bundle maturation with age. In conclusion, the Mahalanobis approach finely described maturational asynchrony across the three SLF branches and confirmed that SLF II had the most delayed maturation.

## Discussion

Our study addresses the difficult question of identifying SLF sub-bundles (SLF I, II and III) in the developing brain and quantifying their maturation, which have not been investigated in previous neonatal dMRI studies using the tensor model. Similar fibre morphology and connectivity of the SLF branches between neonatal and adult groups were found for SLF I and III; however, the changes were visible in SLF II. Quantitative comparisons between groups for Mahalanobis distances also supported the less advanced maturation of SLF II.

### Comparisons of trajectories of SLF branches between the neonatal and adult groups

From the segmentation results of the SLF, we found that the cortical regions connected by the SLF three branches in the human neonatal brain were similar to those of adults, which to some extent verified the accuracy of our segmentation. At 30–31 GW, with the transformation of radial glial fibres to astrocytes, the transition from high angular resolution diffusion imaging (HARDI)-defined radial coherence to corticocortical coherence began simultaneously, indicating the emergence of corticocortical association fibres (Xu et al. 2014; Wilson et al. 2021). Additionally, a study of foetal post-mortem dissection has shown that the parietal portion of the SLF is visible and multi-layered at 32–35 GW (Horgos et al. 2020), and the SLF III segment of the neonatal group in our research also terminated in the supramarginal gyrus, consistent with the trajectory results of the SLF III segment in adults. However, it is noteworthy that the streamlines of SLF II did not reach the parietal areas, such as the inferior parietal lobule and angular gyrus, in the neonatal group, which differed from that in adults. Some important functions that SLF III is often involved in combination with SLF II are tool use, spatial awareness and social learning (Nakajima et al. 2020). Thus, fibre terminations in the parietal cortex of SLF II should have also started to grow and can be observed in infants, similar to the situation for SLF III. However, SLF II was more affected by fibres of the corona radiata than SLF III, and we suspect that the rapid maturation of the corticospinal tract may hinder the accurate observation of SLF II through crossings in the corona radiata (Schurr et al. 2020). In addition, the low myelination of the SLF in the neonatal brain also interfered with the fibre-tracking results, although we used advanced HARDI imaging data. The fibres of SLF II were likely affected due to its low maturity.

Researchers have found that the key starting time points (32 weeks post-conception) for the invasion of corticocortical axons into the cortical plate coincide with the intensive growth of dendrites of layer III pyramidal cells (Petanjek et al. 2008), indicating the important role of layer III neurons in corticocortical connections. Moreover, areas 9 and 46 (middle part of middle frontal gyrus or dorsolateral prefrontal cortex) were where the SLF II component mainly passed through during the neonatal period according to the cytoarchitectonic definition (Rajkowska and Goldman-Rakic 1995). Thus, pyramidal cells of layer III in areas 9 and 46 may play an important role in the SLF II component. Circuits associated with layer IIIC neurons have the most protracted period of developmental plasticity (Petanjek et al. 2019), supporting the selective and protracted maturation of SLF II observed in this study. These unique protracted developmental patterns for associative layer III neurons in the human dorsolateral prefrontal cortex provide a window of opportunity for pathological events to disrupt the normal formation of cognitive circuits involving layer III neurons. Developmental data from Petanjek et al. (Petanjek et al. 2008, 2011) stressed the role of layer III neurons as possible alterations during development for the appearance of autism (Petanjek et al. 2019) and schizophrenia (Arnsten et al. 2022). Collectively, these findings indicate that close attention should be given to the abnormal alterations in layer III neurons in the human dorsolateral prefrontal cortex connected by SLF II during development, which may result in some psychiatric disorders and states in the future.

### Microstructural changes in SLF branches with increasing age at scan

None of the SLF branches showed age-related changes in the mean DTI and NODDI metrics in the adult group. These results make sense, on the one hand, and suggest that the SLF branches may have matured or peaked during this young adult period; therefore, measurements of the SLF reached a plateau and did not vary with age. On the other hand, it is reasonable to consider the Mahalanobis distances between the neonatal and reference adult groups as the estimation method for determining fibre maturity.

We also performed Pearson's correlation analysis of the NODDI and DTI parameter values in relation to PMA in the neonatal group. Our results showed that the FA values of SLF I, SLF II and SLF III all increased linearly with increasing PMA. Many cellular changes are thought to make important contributions to increasing FA, such as the increased density of axonal packing, axonal diameters, axon coherence, changed cell membrane permeability and greater myelination (Jones et al. 2013; Garic et al. 2021). In contrast, the increasing crossing fibres during brain development may result in diffusion signal contamination and a drop in FA (Zhang et al. 2012; Lynch et al. 2020; Kuehn et al. 2022). All of these results indicate that FA is sensitive to age-related changes in microstructure during development but lacks biological specificity. To further explain the increase in FA, we observed changes in the NODDI parameters. The NDI represents the intracellular volume fraction and is often considered the axonal density, while the ODI represents the neurite orientation dispersion, referring to the degree of axonal angular variation in WM (Dean et al. 2017; Genc et al. 2017), probing coherence and geometry. Therefore, increasing NDI or decreasing ODI can lead to an increase in FA (Chang et al. 2015). Our results showed strong positive correlations between the PMA and NDI of all the SLF branches. However, no branches of the SLF had significant correlations between the ODI and PMA. No significant ODI-age correlations in most of the WM tracts during the development period have been observed in previous NODDI studies (Lebel and Deoni 2018; Lebel et al. 2019; Lynch et al. 2020). Together, these results suggest that the increase in FA of the SLF is dominated by an increase in the NDI rather than a constant ODI throughout the neonatal period. The changes in these parameters may reflect that the maturation of the SLF subcomponents was mainly driven by progressive increases in axon density and not accompanied by changes in geometric complexity during this period.

In addition, other DTI parameters (MD, RD and AD) changed differently in the three fibre branches: with the increase in PMA, the MD and RD values of the SLF subcomponents decreased linearly. For AD, there was no significantly correlation with PMA after Bonferroni correction. Among these parameters, the changes in AD are sensitive to axon straightening and tortuosity during development (Lebel and Deoni 2018; Lebel et al. 2019; Goddings et al. 2021), and RD is usually considered to provide particular sensitivity to myelination (Song et al. 2005; Dubois et al. 2008). However, myelination is not the only driver of RD changes. Some neurobiological processes, such as changes in axonal packing and diameter, can also cause changes in RD. In fact, compared with AD and RD, MD is less specific to microstructure (Li et al. 2022). Given our results, we speculated that the decrease in MD in the SLF branches was mainly influenced by the decrease in RD because AD had no age-related changes in all of the SLF branches, which means that some microstructural changes prevented the dispersion of water molecules perpendicular to the fibre direction and led to the decrease in MD during this period.

### Microstructural comparisons of SLF branches between the neonatal and adult groups

To evaluate the maturational degree of the SLF branches in the neonatal period, we used the DTI and NODDI metrics of the adult group as a reference and compared the differences between the neonatal and adult groups. Our study has shown that the strong increase or decrease in parameter values from neonates to young adulthood is widespread across the SLF branches and has different magnitudes for different branches. These large between-group differences suggest that the microstructure of the SLF in the neonatal group may need long-lasting maturation, which is consistent with the idea that the SLF may contribute to higher-order brain functions (Grinberg et al. 2017; Ouyang et al. 2019). To further quantify the magnitude of changes in age-related DTI and NODDI measures between the two groups, we calculated Cohen's d effect size. Among these parameters, the d_ODI_ of each SLF branch was relatively small, indicating that the differences in the ODI between the neonatal and adult groups were not large. Lynch et al. observed that the ODI of most WM tracts did not show significant associations with age from infancy through adolescence (0.6–18.8 years), suggesting that the development of WM over this time period may be accompanied by an unchanged ODI corresponding to the geometric complexity of axons (Lynch et al. 2020). In addition, the ODI is sensitive to various neurobiological processes, which have both positive and negative contributions to the ODI, such as bending and fanning of axons, crossing fibres, glial infiltration and neurite pruning and obscure any gross changes in the ODI (Genc et al. 2017). These findings need further research and discussion.

Some important hallmarks of WM maturation after birth are increased packing, calibre and myelination of the axonal pathways (LaMantia and Rakic 1990; Kunz et al. 2014; Lynch et al. 2020), which can be indirectly detected by DTI measures. However, these measures cannot distinguish the specific biological process of microstructural development. NDI in multi-compartment NODDI model was largely invariant to fibre crossing angle, which may enable the investigation of the contributing tissue components individually, and contributes to the explanation of DTI parameters (Genc et al. 2017; Kuehn et al. 2022). Nevertheless, the complexity of white matter maturation cannot be fully delineated by one of these parameters alone because different parameters are sensitive to different tissue properties (Kulikova et al. 2015). Thus, we chose multi-parametric Mahalanobis approach to measure the maturity of SLF branches of neonates by integrating all NODDI and DTI metrics, which provide a measure of the maturational distance between neonatal and adult brains. There was no statistically significant difference in Mahalanobis distance between SLF I and SLF III, indicating no difference in maturity. SLF II exhibited the largest Mahalanobis distance and thus may be the least mature of the three branches. The ventral SLF III was more mature than the dorsal SLF II according to their Mahalanobis distances. Dubois et al. also observed similar developmental tempos in linguistic bundles: the maturation of the ventral pathway was more advanced than that of the dorsal pathway in the periinsular area (Dubois et al. 2016). Additionally, Horgos and colleagues found that newer fibres always emerge in more superficial positions with increasing PMA and form a more dorsal component of the SLF (Horgos et al. 2020), indicating the later appearance of the dorsal SLF II segment than the ventral SLF III.

The developmental tempo of WM tracts is usually consistent with the level of brain functions they undertake. The WM tracts that mature earlier may be involved in the basic functions for life. Conversely, the later-matured tracts may contribute more to higher-level brain functions (Ouyang et al. 2019). Parlatini et al. found that the functions of the human frontal and parietal regions can be divided into dorsal spatial/motor and ventral nonspatial/motor networks, corresponding to the SLF I and SLF III functional networks, respectively. However, SLF II is associated with a network of multi-modal regions at the intersection between the dorsal and ventral networks. There may be many neurons with very flexible response characteristics in the regions connected by SLF II, reflecting the function of human conscious processing (Parlatini et al. 2017). In addition, some studies have suggested that SLF I mainly connects the core areas of the dorsal attention network (DAN), and SLF III mainly connects the areas of the ventral attention network (VAN). The middle SLF II, however, was reported to directly connect the DAN and the VAN, which may provide critical anatomical communication for the two attention systems (Thiebaut De Schotten et al. 2011; Sani et al. 2019; Suo et al. 2021). Therefore, the later development tracts, such as SLF II, may contribute more to higher-level brain functions, especially in mediating between SLF I and SLF III.

### Limitations and future directions

We segmented the neonatal SLF into three branches and quantitatively assessed the maturation status of each branch by comparing diffusion parameters with those of adults. None of this has been deeply explored before. However, one of the limitations of our study is that tractography ROIs were manually defined because of the marked individual variations, which is time consuming; thus, a more automated method is required for future work. In addition, although dMRI tractography is currently the only non-invasive method for detecting the WM structure in vivo, our results need to be histologically verified for the widely acknowledged limitations of fibre tracking, such as tractography dissection variability and heterogeneity (Schilling et al. 2021). We will supplement and confirm our studies in the future through the fibre dissection technique in newborn specimens.

## Conclusions

The fibre morphology and connectivity of SLF sub-bundles were revealed in our study. We observed similar trajectories of the SLF branches between the neonatal and adult groups except that the streamlines of SLF II in the neonatal group did not reach the angular gyrus, likely due to its low degree of myelination, which is different from that in adults. Abnormal alterations in layer III neurons in the human dorsolateral prefrontal cortex connected by SLF II during development may result in some psychiatric disorders and states and deserve more attention in the future. In addition, the quantitative results showed that SLF II had the highest Mahalanobis distance and was the least mature subcomponent, indicating that it may be involved in more advanced brain functions. Our results may provide new reference indicators for the early diagnosis and treatment of diseases related to the abnormal development of SLF sub-bundles in the neonatal period.

## Supplementary Information

Below is the link to the electronic supplementary material.

Supplementary file1 (DOCX 6779 KB)

## Data Availability

The data for 40 normal term-born neonates were acquired from Developing Human Connectome Project (dHCP) (http://developingconnectome.org/) and 40 healthy young adults (ages 22–35 years) from Human Connectome Project (HCP) (https://www.humanconnectome.org).

## Code availability

Our Python script developed for calculating the mean metric value over the tract can be downloaded from https://data.mendeley.com/datasets/5yymds8v5b/1.
