# Supplementary material for: A comparative study of the superior longitudinal fasciculus subdivisions between neonates and young adults
Source: Brain Struct Funct. 2022 Sep 17;227(8):2713–30. doi: 10.1007/s00429-022-02565-z (PMC9618541; doi:10.1007/s00429-022-02565-z)
Supplement: Supplementary file 1 — Supplementary file1 (DOCX 6779 KB) [file 429_2022_2565_MOESM1_ESM.docx]

**Supplementary Online Content**

**Supplementary Figure 1.** WM-FOD map in axial(A), sagittal(B) and coronal(C) view

**Supplementary Figure 2.** Delineation of inclusion and exclusion ROIs used for segmentation of left and right SLF three branches, overlaid on a synthetic T1-weighted image in a representative participant of adult group

**Supplementary Figure 3.** Cortical ROIs based on the neonatal AAL template

**Supplementary Figure 4.** NODDI and DTI metrics' values of whole brain

**Supplementary Figure 5.** Hemispheric lateralization of each SLF branch (SLF I, II, and III) in neonatal group assessed by the lateralization index (LI) of NODDI and DTI metrics

**Supplementary Figure 6.** The scatter plot of tract-specific NDI values varying with post-menstrual age

**Supplementary Figure 7.** The scatter plot of tract-specific ODI values varying with post-menstrual age

**Supplementary Figure 8.** The scatter plot of tract-specific FA values varying with post-menstrual age

**Supplementary Figure 9.** The scatter plot of tract-specific MD values varying with post-menstrual age

**Supplementary Figure 10.** The scatter plot of tract-specific AD values varying with post-menstrual age

**Supplementary Figure 11.** The scatter plot of tract-specific RD values varying with post-menstrual age

**Supplementary Table 1.** Pearson’s correlation coefficient (r) values and corresponding p values between diffusion metrics and post-menstrual age in various SLF branches among neonates.

**Supplementary Table 2a.** Mean values (M) and standard deviations (SD) of NODDI and DTI parameters in various SLF branches for neonates.

**Supplementary Table 2b.** Mean values (M) and standard deviations (SD) of NODDI and DTI parameters in various SLF branches for adults.

**Supplementary Table 3.** Absolute Cohen's d values of NODDI and DTI parameters for various SLF branches quantifying the differences of metrics between neonatal and adult groups


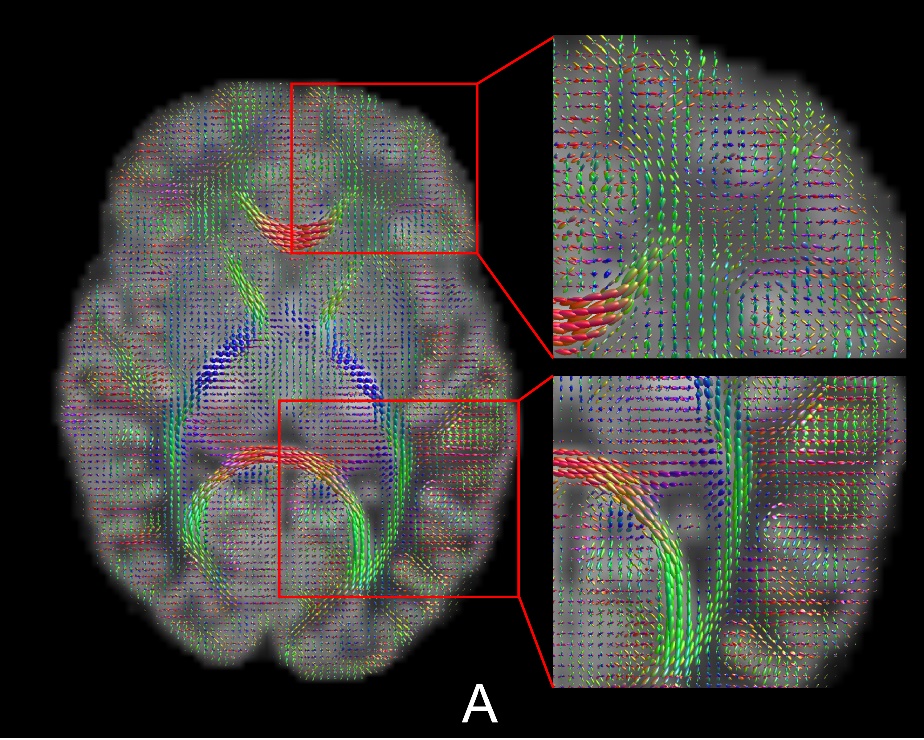


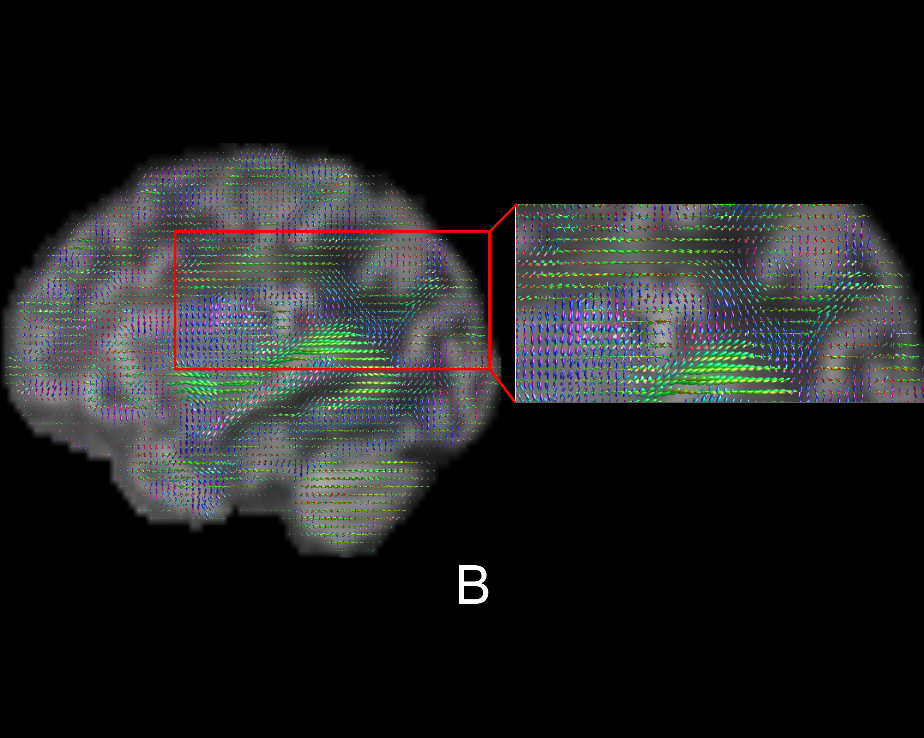


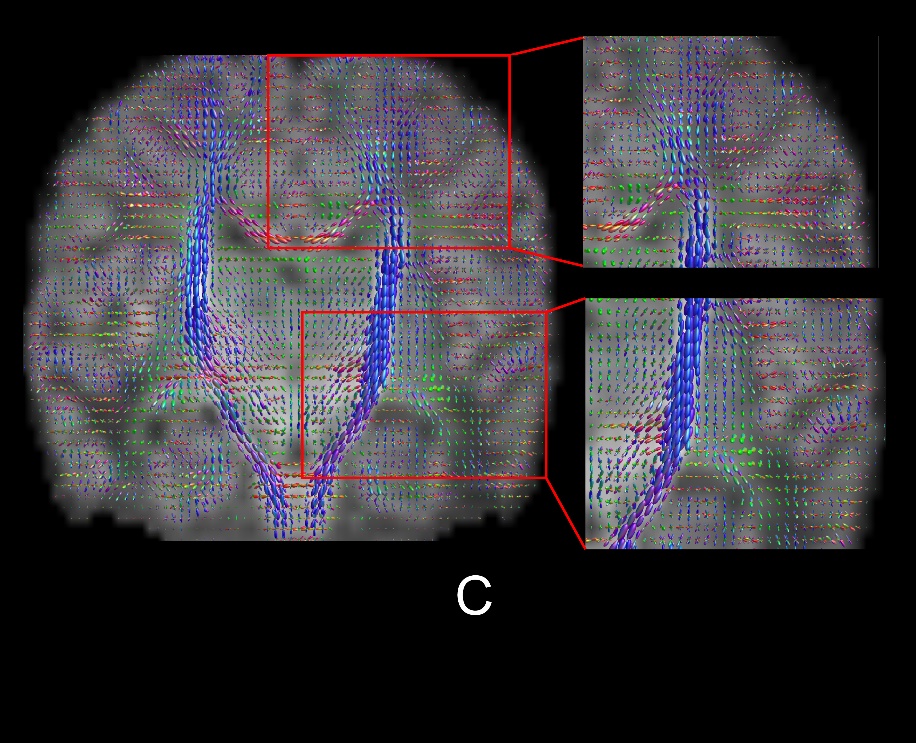


**SM-Fig. 1.** WM-FOD map in axial(A), sagittal(B) and coronal(C) view.


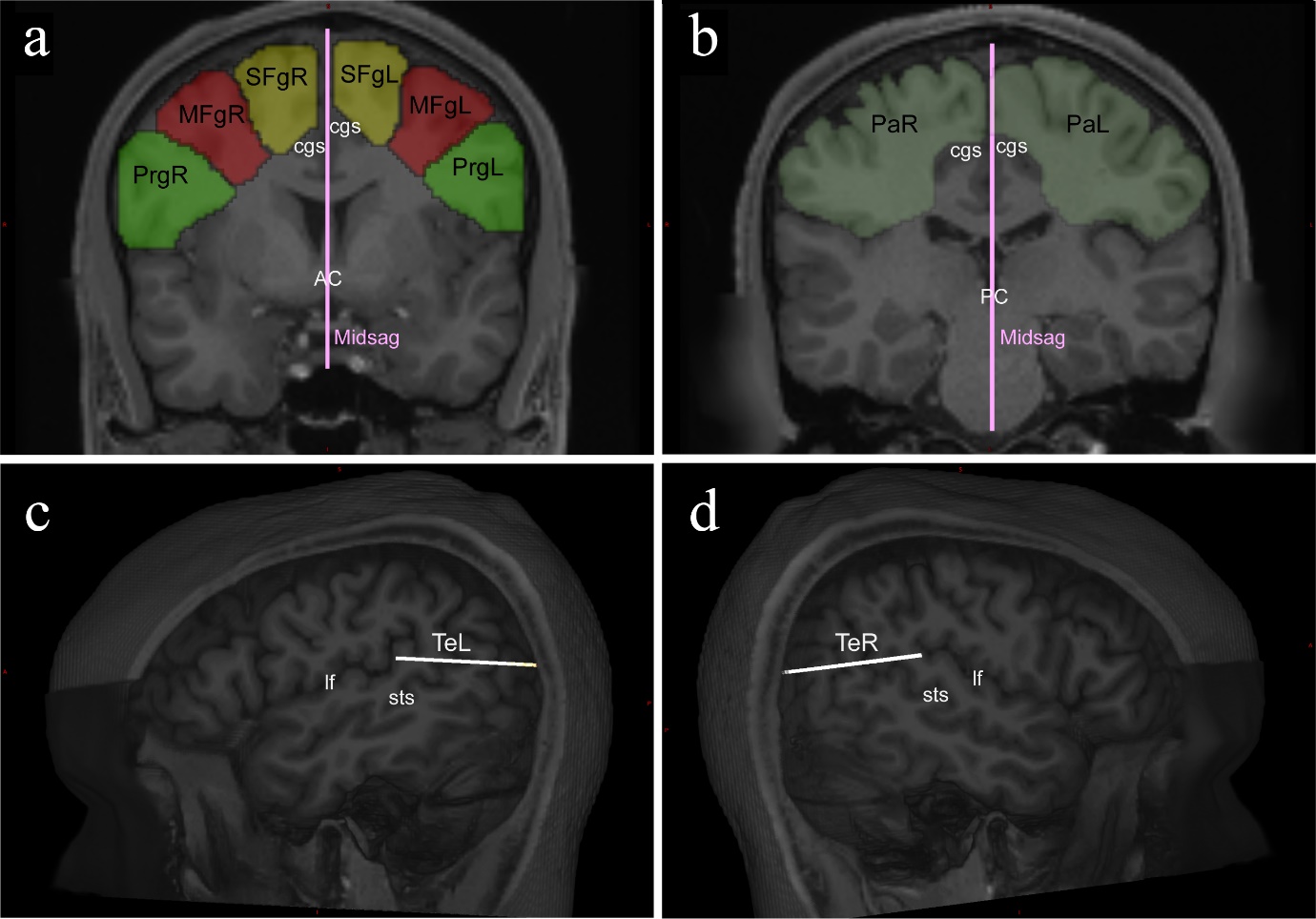


**SM-Fig. 2.** Delineation of inclusion and exclusion ROIs used for segmentation of left and right SLF three branches, overlaid on a synthetic T1-weighted image in a representative participant of adult group. **(a).** Coronal ROIs in the left and right frontal lobes. **(b).** Coronal ROIs in the left and right parietal lobes. **(c-d).** Axial ROIs in the left and right hemispheres used to exclude the streamlines of arcuate fasciculus projecting to the temporal lobe. **Abbreviations: SFgL** superior frontal gyrus left, **SFgR** superior frontal gyrus right, **MFgL** middle frontal gyrus left, **MFgR** middle frontal gyrus right, **PrgL** precentral gyrus left, **PrgR** precentral gyrus right, **PaL** parietal left, **PaR** parietal right, **TeL** temporal left, **TeR** temporal right, **AC** anterior commissure, **PC** posterior commissure, **Midsag** mid-sagittal plane, **cgs** cingulate sulcus, **sts** superior temporal sulcus, **lf** lateral fissure.

‘AND’ ROIs ‘NOT’ ROIs ROIs selected for tractography

PaL TeL left SLF I: PaL ‘AND’ SFgL ‘NOT’ TeL ‘NOT’ Midsag

PaR TeR left SLF II: PaL ‘AND’ MFgL ‘NOT’ TeL ‘NOT’ Midsag

SFgL Midsag left SLF III: PaL ‘AND’ PrgL ‘NOT’ TeL ‘NOT’ Midsag

SFgR right SLF I: PaR ‘AND’ SFgR ‘NOT’ TeR ‘NOT’ Midsag

MFgL right SLF II: PaR ‘AND’ MFgR ‘NOT’ TeR ‘NOT’ Midsag

MFgR right SLF III: PaR ‘AND’ PrgR ‘NOT’ TeR ‘NOT’ Midsag

PrgL

PrgR

**SM-Fig. 3.**
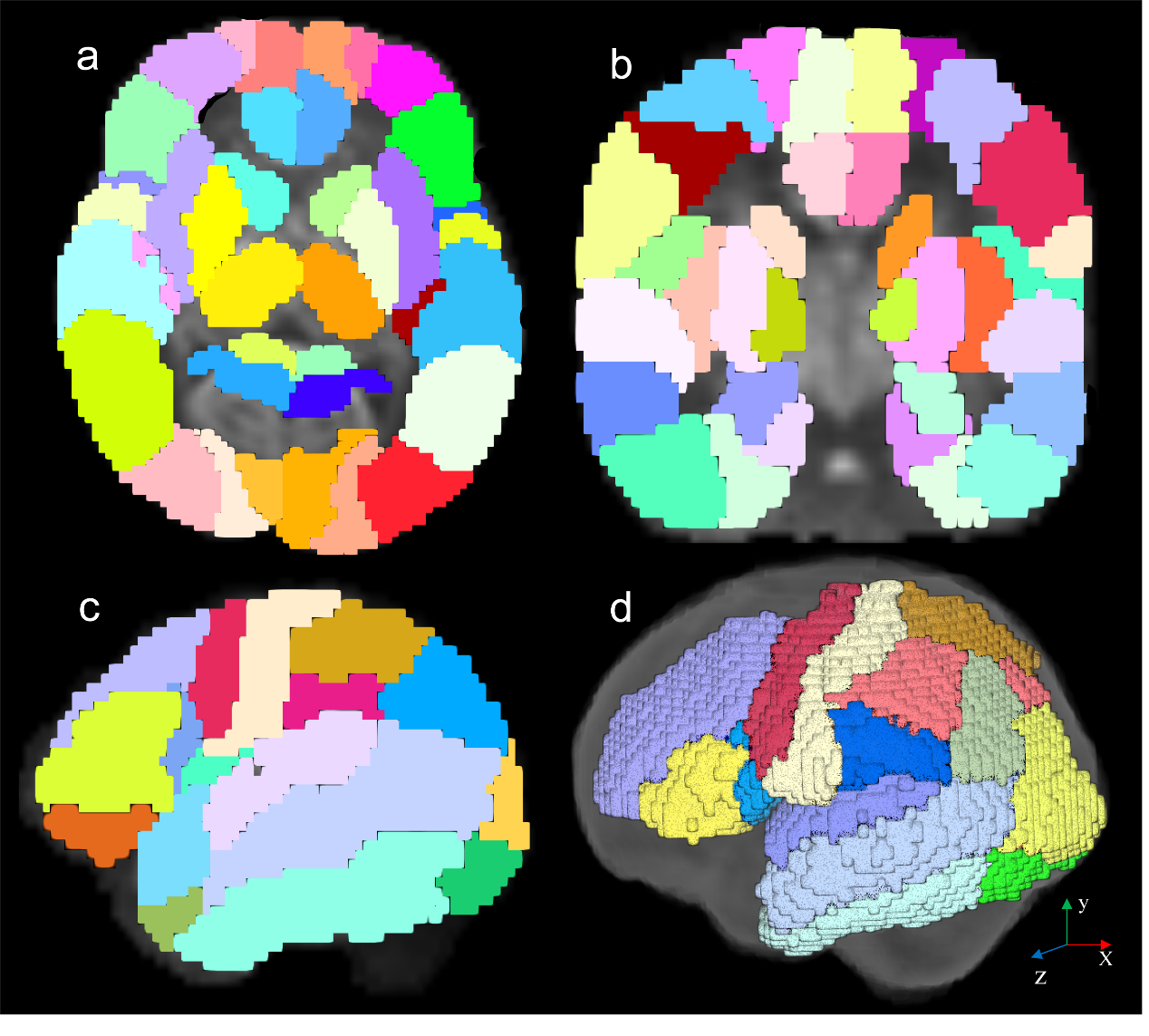
 Cortical ROIs based on the neonatal AAL template. **(a-c).** 90 anatomical regions of the brain in axial, coronal and sagittal positions, with different colors representing different anatomical regions. **(d).** a three-dimensional view of some ROIs.


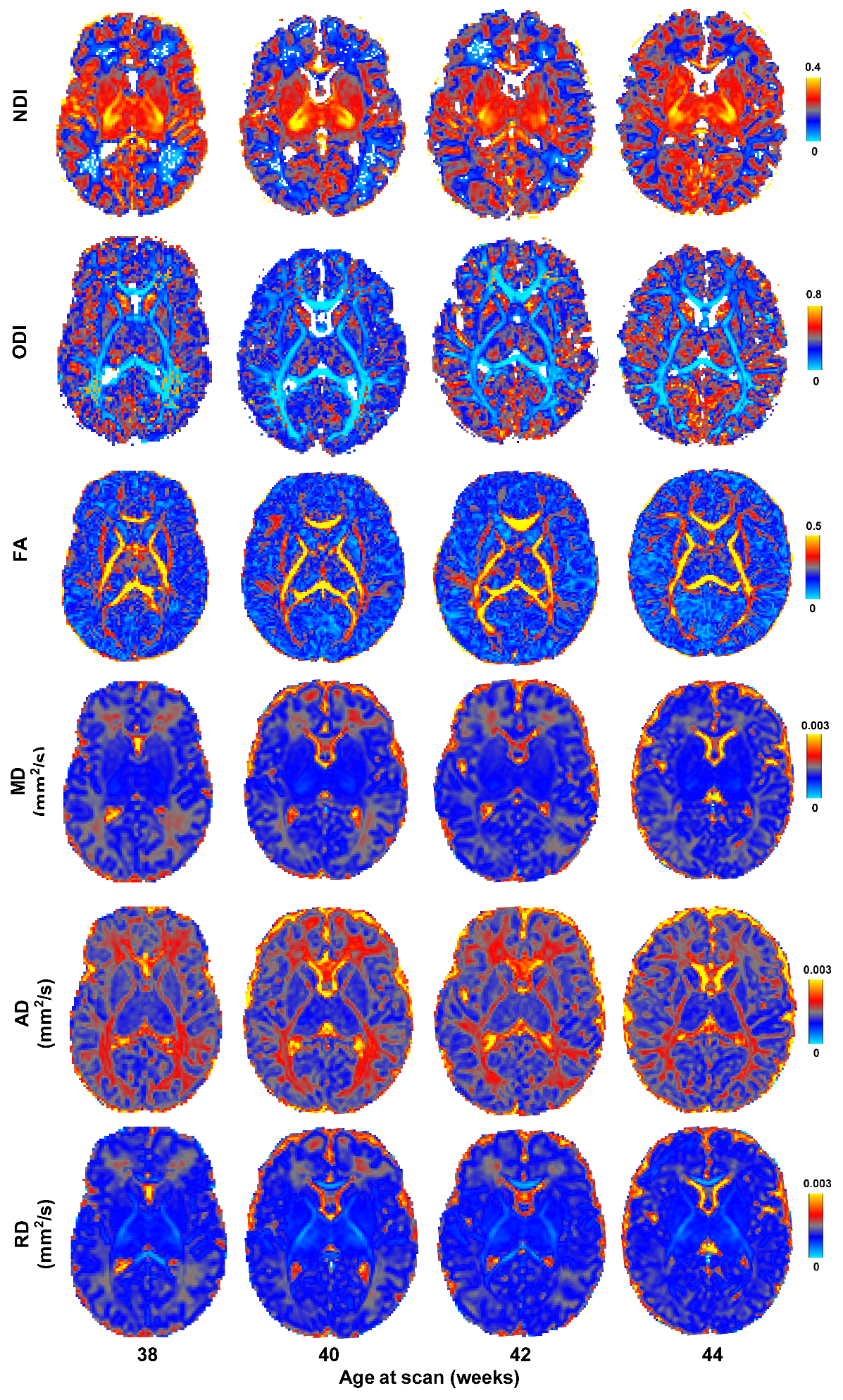


**SM-****Fig. 4.** NODDI and DTI metrics' values of whole brain. We selected four neonatal individuals of different ages for the presentation.


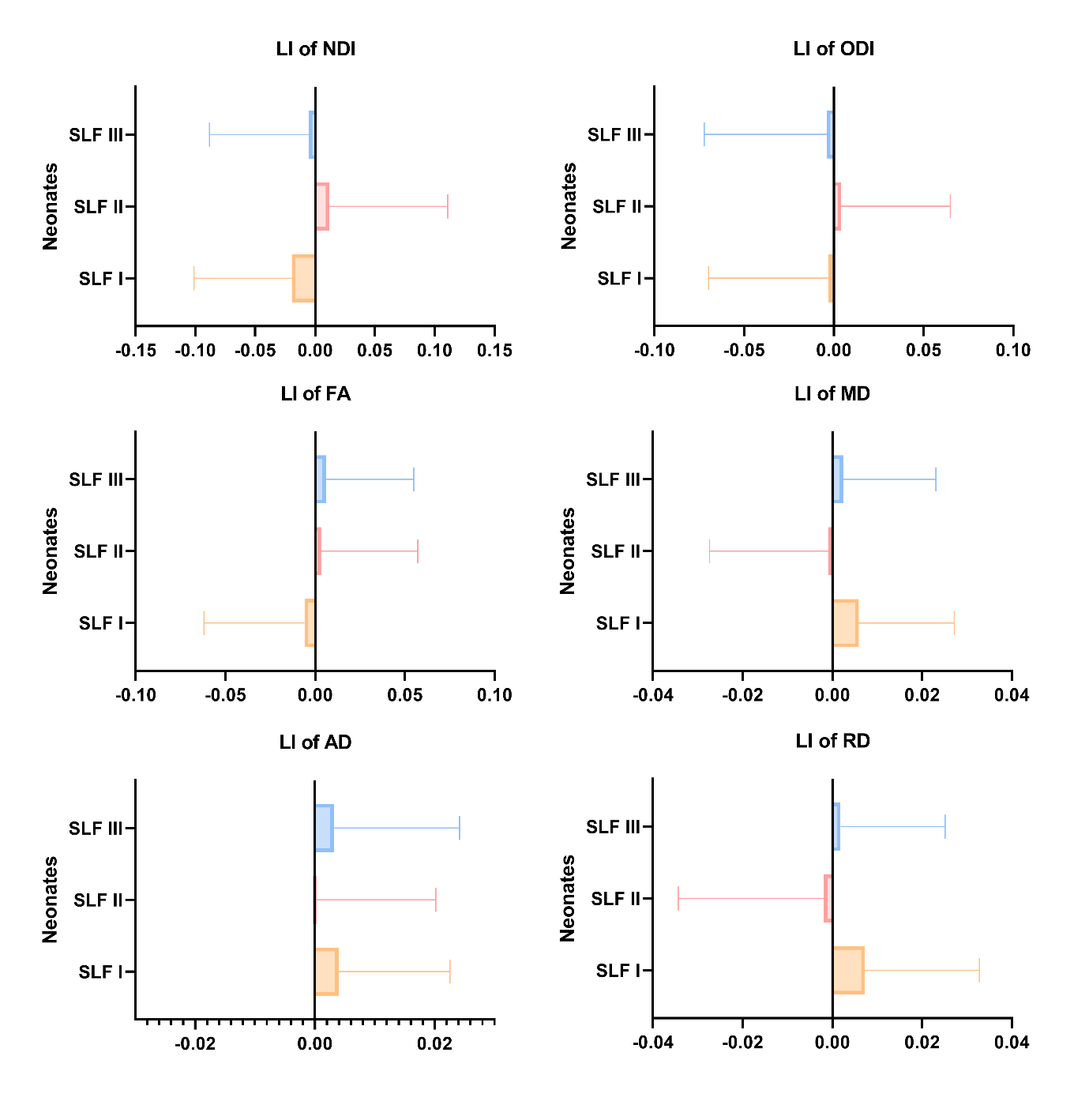


**SM-Fig. 5.** Hemispheric lateralization of each SLF branch (SLF I, II, and III) in neonatal group assessed by the lateralization index (LI) of NODDI and DTI metrics. A positive LI value indicates a left lateralization, and a negative LI value indicates a right lateralization. There were no statistically significant differences between the left and right hemispheres of the three SLF branches (p >0.05).


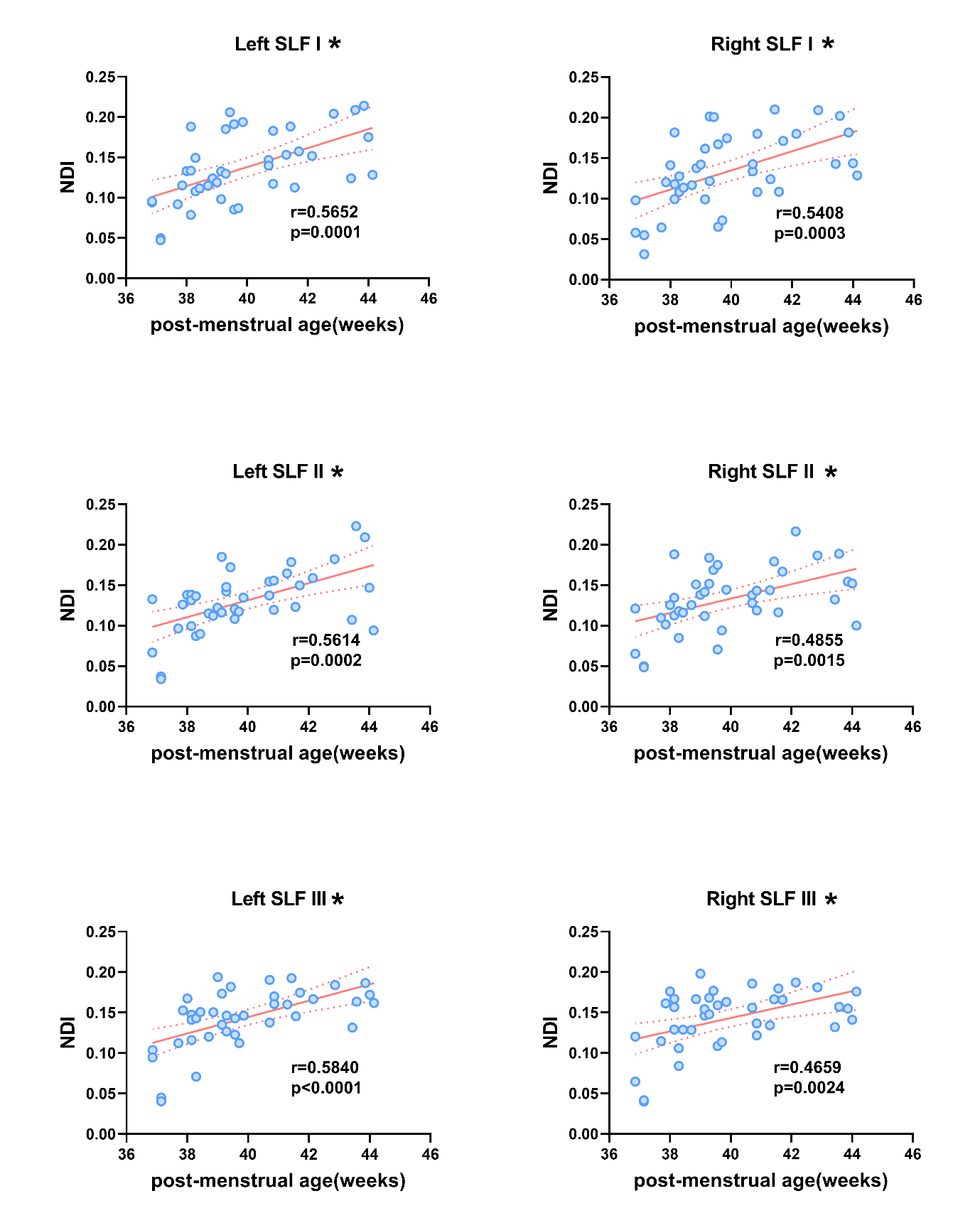


**SM-Fig. 6.** The scatter plot of tract-specific NDI values varying with post-menstrual age. *The significance of correlation analysis remained when Bonferroni-Dunn correction was set at p < 0.008.


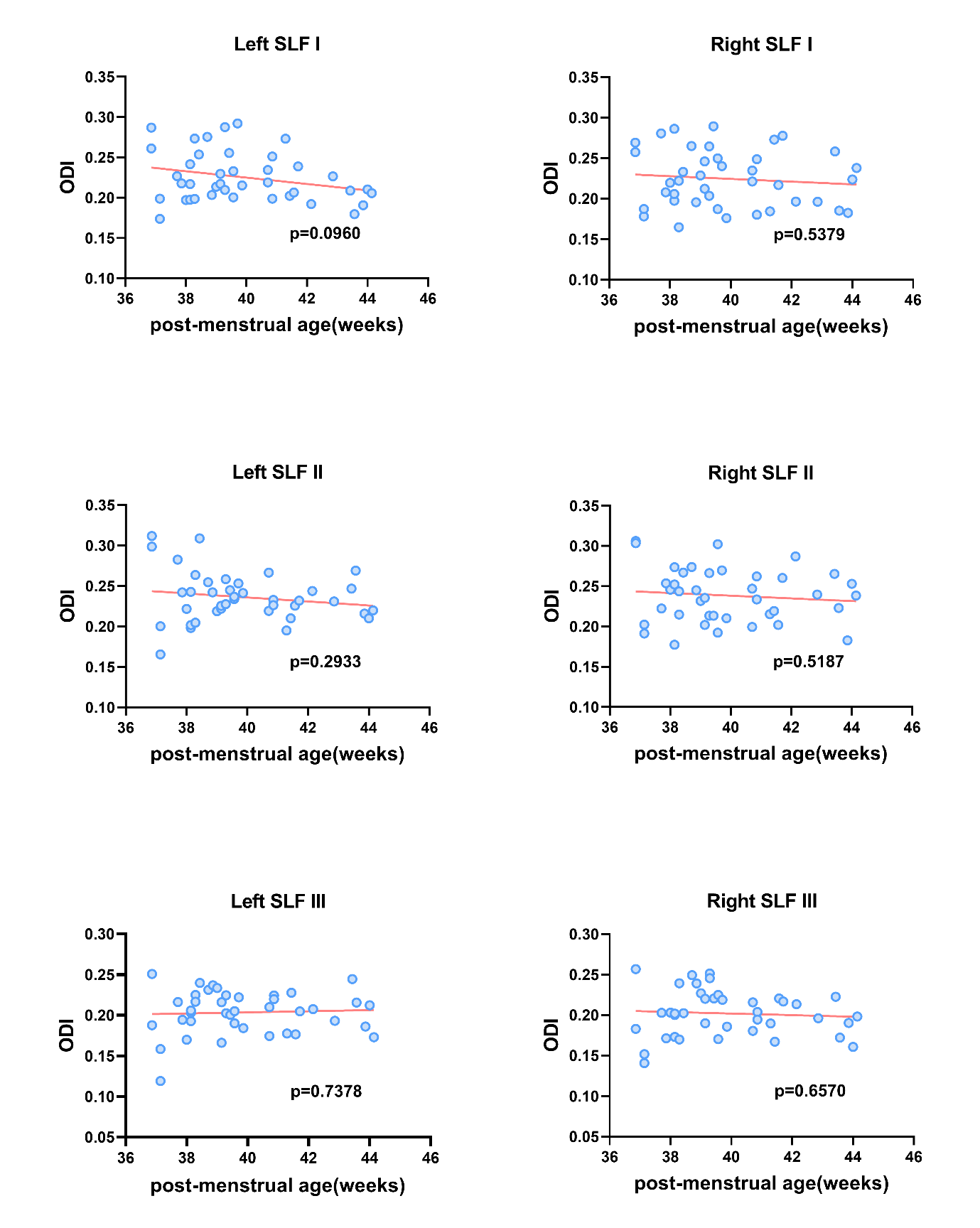


**SM-Fig. 7.** The scatter plot of tract-specific ODI values varying with post-menstrual age.


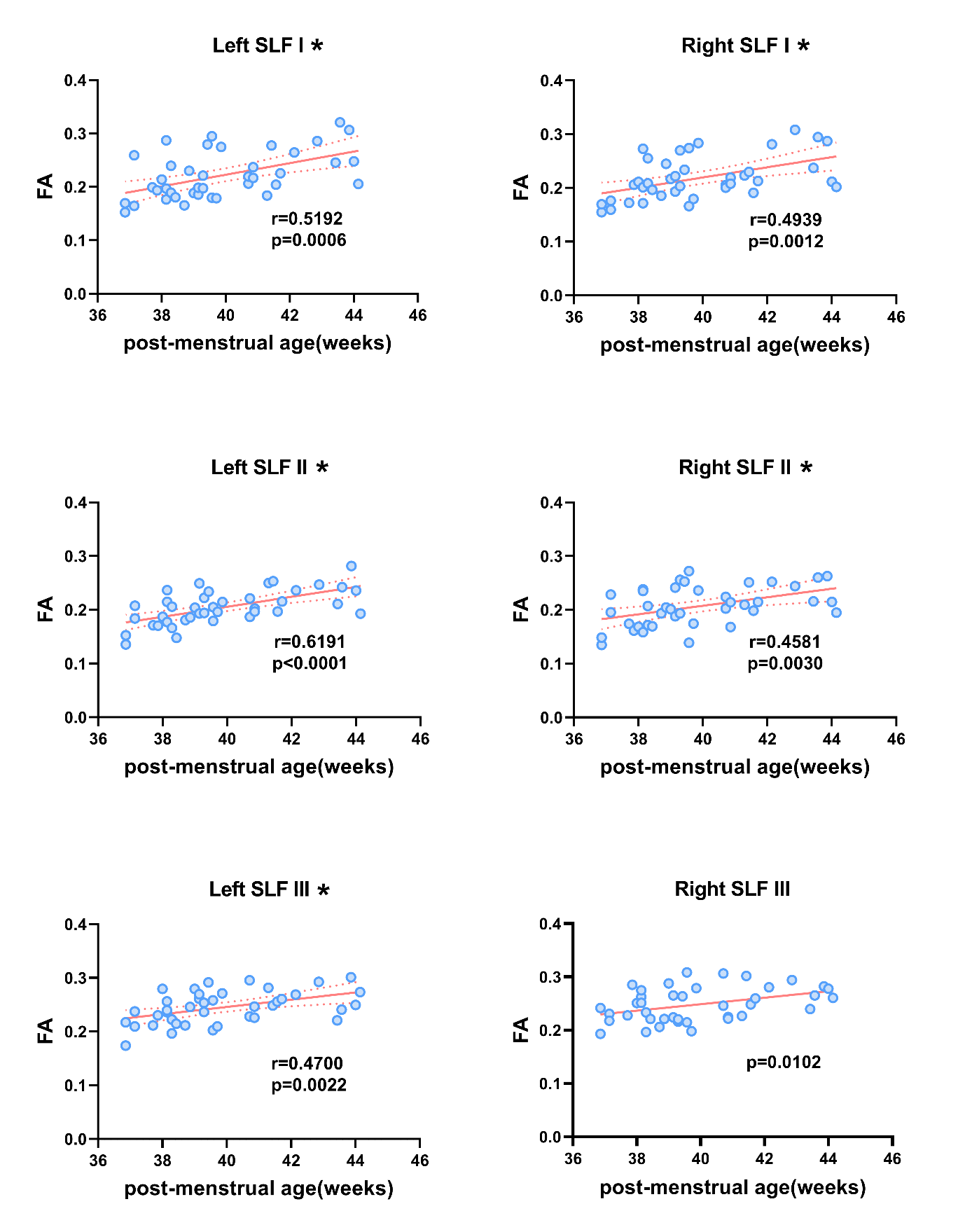


**SM-Fig. 8.** The scatter plot of tract-specific FA values varying with post-menstrual age. *The significance of correlation analysis remained when Bonferroni-Dunn correction was set at p < 0.008.


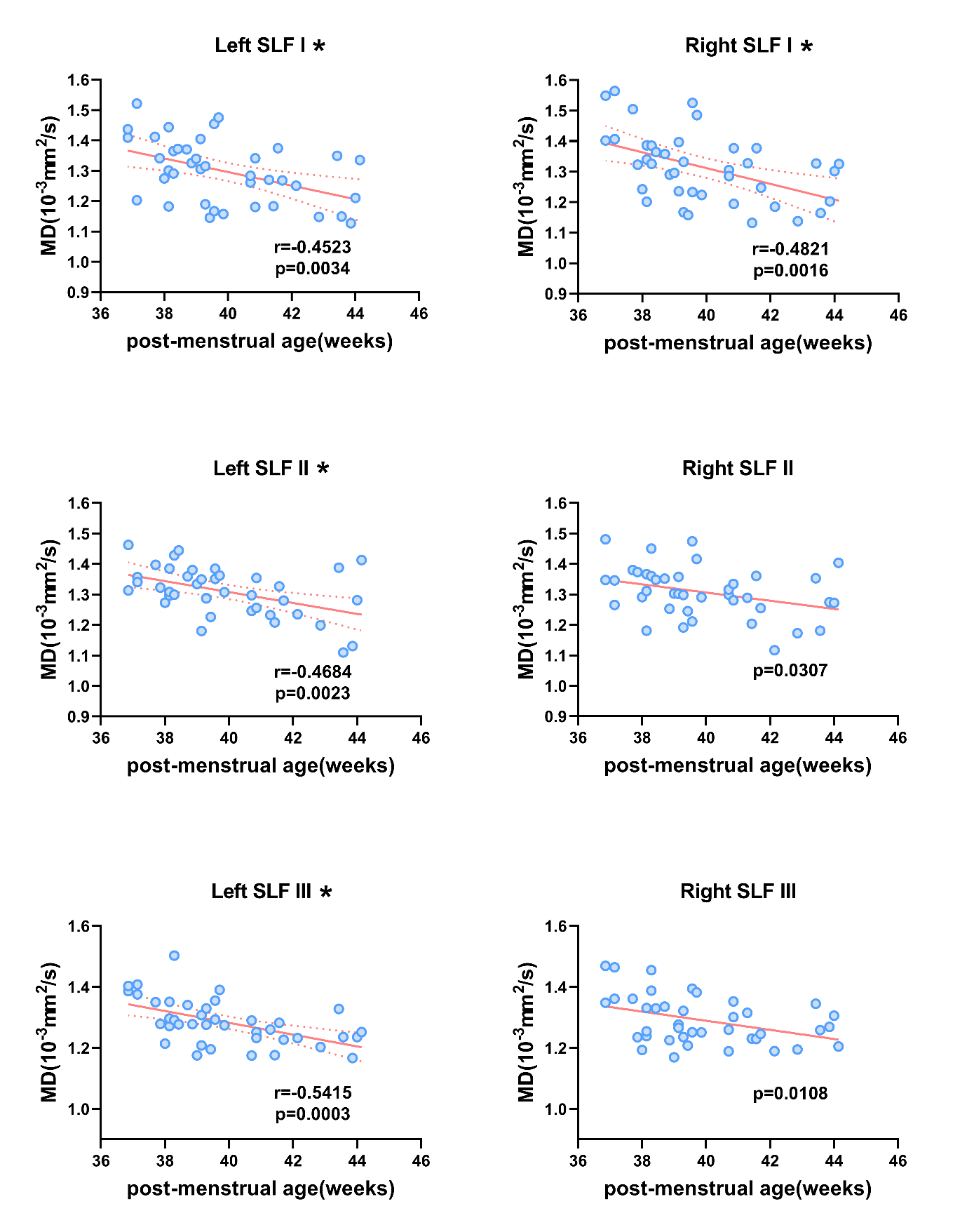


**SM-Fig. 9.** The scatter plot of tract-specific MD values varying with post-menstrual age. *The significance of correlation analysis remained when Bonferroni-Dunn correction was set at p < 0.008.


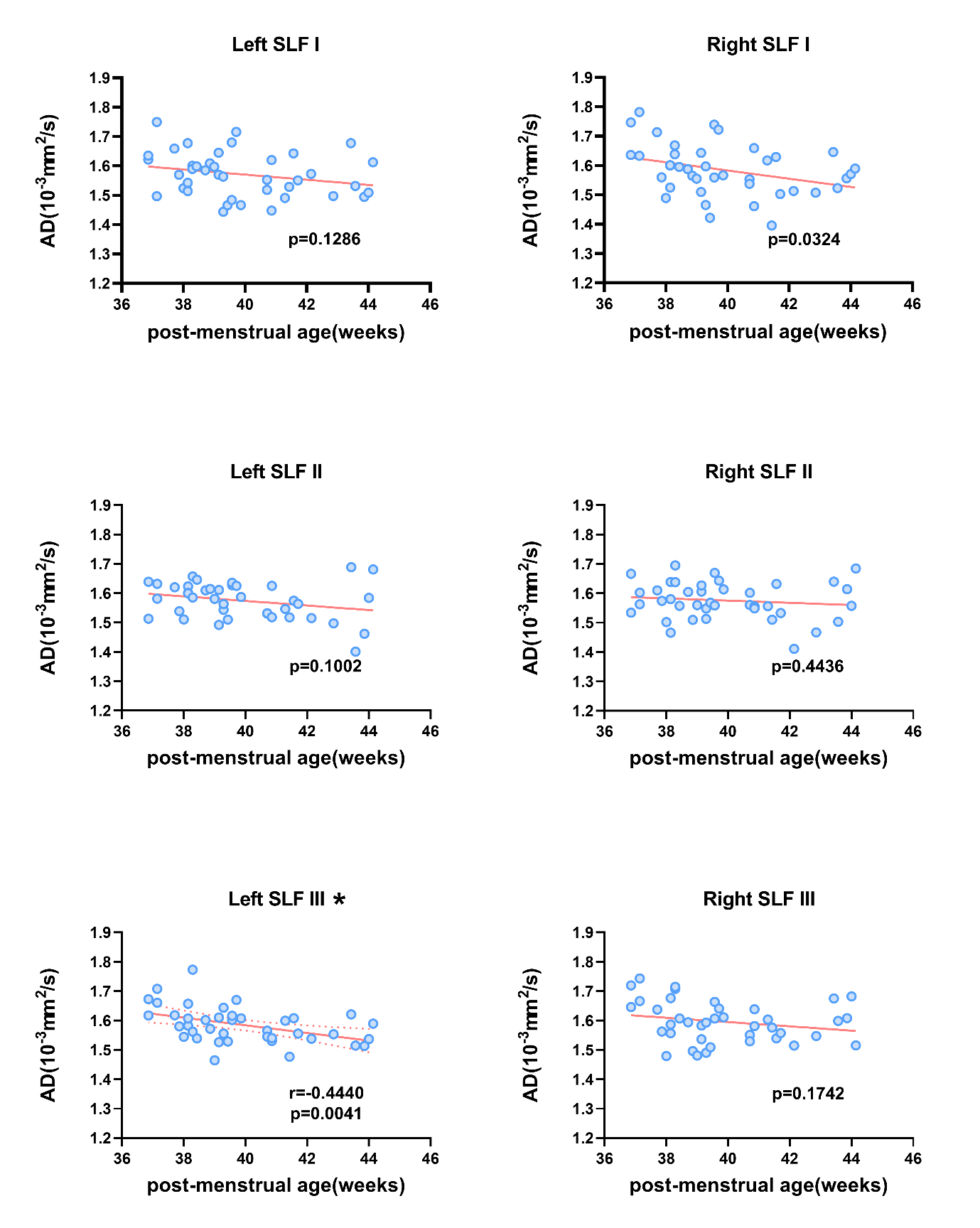


**SM-Fig. 10.** The scatter plot of tract-specific AD values varying with post-menstrual age. *The significance of correlation analysis remained when Bonferroni-Dunn correction was set at p < 0.008.


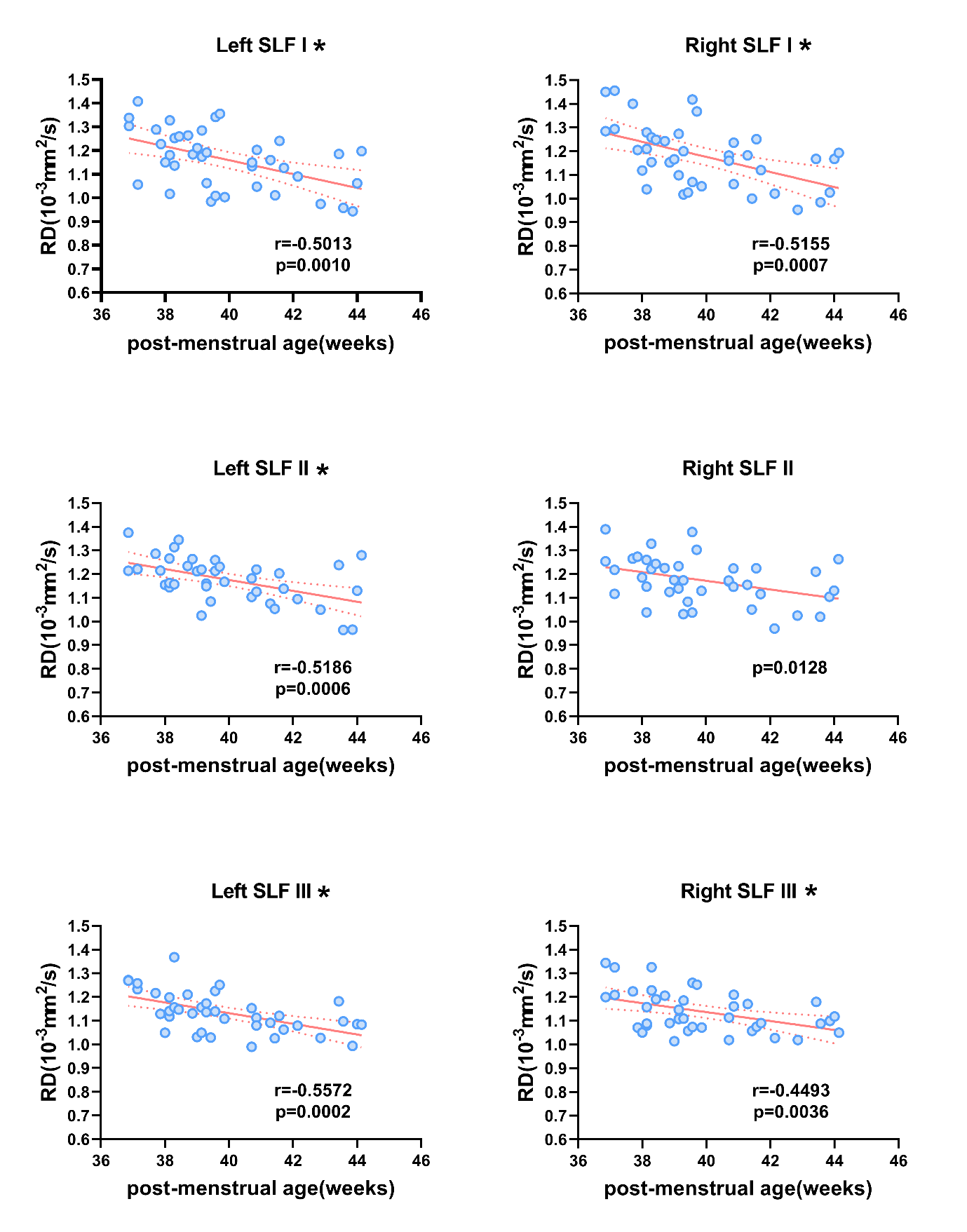


**SM-Fig. 11.** The scatter plot of tract-specific RD values varying with post-menstrual age. *The significance of correlation analysis remained when Bonferroni-Dunn correction was set at p < 0.008.

**SM-Table 1.**

Pearson’s correlation coefficient (r) values and corresponding p values between diffusion metrics and post-menstrual age in various SLF branches among neonates.

|  | | **L_ SLF I** | **R_ SLF I** | **L_ SLF II** | **R_ SLF II** | **L_ SLF III** | **R_ SLF III** |
| --- | --- | --- | --- | --- | --- | --- | --- |
| **NDI** | **r** | 0.5652 | 0.5408 | 0.5614 | 0.4855 | 0.584 | 0.4659 |
|  | **p** | 0.0001**^***^** | 0.0003**^**^** | 0.0002**^**^** | 0.0015**^*^** | <0.0001**^***^** | 0.0024**^*^** |
|  | **t** | 4.2234 | 3.9633 | 4.1819 | 3.4234 | 4.4349 | 3.2458 |
|  | **df** | 38 | 38 | 38 | 38 | 38 | 38 |
| **ODI** | **r** | -0.2668 | -0.1003 | -0.1704 | -0.1051 | -0.0546 | -0.0724 |
|  | **p** | 0.0960 | 0.5379 | 0.2933 | 0.5187 | 0.7378 | 0.6570 |
|  | **t** | -1.7065 | -0.6214 | -1.0660 | -0.6515 | -0.3373 | -0.4475 |
|  | **df** | 38 | 38 | 38 | 38 | 38 | 38 |
| **FA** | **r** | 0.5192 | 0.4939 | 0.6191 | 0.4581 | 0.47 | 0.4018 |
|  | **p** | 0.0006**^**^** | 0.0012**^*^** | <0.0001**^***^** | 0.0030**^*^** | 0.0022**^*^** | 0.0102 |
|  | **t** | 3.7449 | 3.5015 | 4.8597 | 3.1769 | 3.2824 | 2.7048 |
|  | **df** | 38 | 38 | 38 | 38 | 38 | 38 |
| **MD** | **r** | -0.4523 | -0.4821 | -0.4684 | -0.342 | -0.5415 | -0.3988 |
|  | **p** | 0.0034**^*^** | 0.0016^*^ | 0.0023**^*^** | 0.0307 | 0.0003**^**^** | 0.0108 |
|  | **t** | -3.1262 | -3.3921 | -3.2681 | -2.2435 | -3.9705 | -2.6808 |
|  | **df** | 38 | 38 | 38 | 38 | 38 | 38 |
| **AD** | **r** | -0.2444 | -0.339 | -0.2636 | -0.1246 | -0.4440 | -0.2192 |
|  | **p** | 0.1286 | 0.0324 | 0.1002 | 0.4436 | 0.0041**^*^** | 0.1742 |
|  | **t** | -1.5537 | -2.2213 | -1.6845 | -0.7741 | -3.0546 | -1.3849 |
|  | **df** | 38 | 38 | 38 | 38 | 38 | 38 |
| **RD** | **r** | -0.5013 | -0.5155 | -0.5186 | -0.3903 | -0.5572 | -0.4493 |
|  | **p** | 0.0010**^**^** | 0.0007**^**^** | 0.0006**^**^** | 0.0128 | 0.0002**^**^** | 0.0036**^*^** |
|  | **t** | -3.5714 | -3.7085 | -3.7390 | -2.6132 | -4.1364 | -3.1002 |
|  | **df** | 38 | 38 | 38 | 38 | 38 | 38 |

The significance of the correlation analysis after Bonferroni-Dunn correction was defined as *p<0.008; **p≤0.001; and ***p≤0.0001.

**SM-Table 2a.**

Mean values (M) and standard deviations (SD) of NODDI and DTI parameters in various SLF branches for neonates.

|  | | L_ SLF I | R_ SLF I | L_ SLF II | R_ SLF II | L_ SLF III | R_ SLF III |
| --- | --- | --- | --- | --- | --- | --- | --- |
| **NDI** | **M** | 0.1366 | 0.1335 | 0.1304 | 0.1325 | 0.1434 | 0.1424 |
|  | **SD** | 0.04370 | 0.04589 | 0.03935 | 0.03857 | 0.03648 | 0.03727 |
| **ODI** | **M** | 0.2254 | 0.2247 | 0.2362 | 0.2385 | 0.2035 | 0.2020 |
|  | **SD** | 0.03112 | 0.03520 | 0.03055 | 0.03344 | 0.02661 | 0.02831 |
| **FA** | **M** | 0.2216 | 0.2184 | 0.2046 | 0.2068 | 0.2451 | 0.2482 |
|  | **SD** | 0.04379 | 0.04020 | 0.03143 | 0.03624 | 0.03006 | 0.03178 |
| **MD**  **(10^-3^mm^2^/s)** | **M** | 1.298 | 1.314 | 1.310 | 1.308 | 1.284 | 1.291 |
|  | **SD** | 0.1043 | 0.1129 | 0.08049 | 0.08256 | 0.07510 | 0.07890 |
| **AD**  **(10^-3^mm^2^/s)** | **M** | 1.571 | 1.584 | 1.575 | 1.575 | 1.585 | 1.596 |
|  | **SD** | 0.07538 | 0.08695 | 0.06215 | 0.06291 | 0.06173 | 0.07007 |
| **RD**  **(10^-3^mm^2^/s)** | **M** | 1.162 | 1.179 | 1.178 | 1.174 | 1.134 | 1.138 |
|  | **SD** | 0.1227 | 0.1298 | 0.09319 | 0.09848 | 0.08489 | 0.08796 |

**SM-Table 2b.**

Mean values (M) and standard deviations (SD) of NODDI and DTI parameters in various SLF branches for adults.

|  | | L_ SLF I | R_ SLF I | L_ SLF II | R_ SLF II | L_ SLF III | R_ SLF III |
| --- | --- | --- | --- | --- | --- | --- | --- |
| **NDI** | **M** | 0.6935 | 0.7019 | 0.6924 | 0.7085 | 0.7419 | 0.7629 |
|  | **SD** | 0.02557 | 0.02966 | 0.02657 | 0.02344 | 0.03736 | 0.03499 |
| **ODI** | **M** | 0.2416 | 0.2445 | 0.2968 | 0.2891 | 0.2564 | 0.2594 |
|  | **SD** | 0.01942 | 0.01851 | 0.01722 | 0.02000 | 0.01300 | 0.01612 |
| **FA** | **M** | 0.4754 | 0.4801 | 0.4058 | 0.4165 | 0.4793 | 0.4667 |
|  | **SD** | 0.03047 | 0.02956 | 0.03010 | 0.03244 | 0.03357 | 0.03169 |
| **MD**  **(10^-3^mm^2^/s)** | **M** | 0.7669 | 0.7479 | 0.7491 | 0.7560 | 0.7428 | 0.7594 |
|  | **SD** | 0.02969 | 0.02812 | 0.03598 | 0.04039 | 0.04293 | 0.04072 |
| **AD**  **(10^-3^mm^2^/s)** | **M** | 1.183 | 1.158 | 1.090 | 1.113 | 1.141 | 1.156 |
|  | **SD** | 0.03941 | 0.03789 | 0.04252 | 0.05254 | 0.04521 | 0.04705 |
| **RD**  **(10^-3^mm^2^/s)** | **M** | 0.5586 | 0.5427 | 0.5789 | 0.5777 | 0.5434 | 0.5611 |
|  | **SD** | 0.03513 | 0.03305 | 0.03853 | 0.04201 | 0.04576 | 0.04381 |

**SM-Table 3.**

Absolute Cohen's d values of NODDI and DTI parameters for various SLF branches quantifying the differences of metrics between neonatal and adult groups.

|  | **d_NDI_** | **d_ODI_** | **d_FA_** | **d_MD_** | **d_AD_** | **d_RD_** |
| --- | --- | --- | --- | --- | --- | --- |
| **SLF I** | 15.55 | 0.75 | 7.503 | 7.229 | 7.078 | 6.951 |
| **SLF II** | 18.604 | 2.427 | 7.106 | 10.055 | 10.593 | 9.101 |
| **SLF III** | 18.134 | 2.888 | 8.719 | 9.837 | 9.532 | 9.436 |
